# Availability and Accessibility of Orphan Medicinal Products to Patients in Slovakia in the Years 2010–2019

**DOI:** 10.3389/fphar.2022.768325

**Published:** 2022-01-26

**Authors:** Tatiana Foltanova, Alan Majernik, Eva Malikova, Stanislava Kosirova

**Affiliations:** ^1^ Department of Pharmacology and Toxicology, Comenius University in Bratislava, Bratislava, Slovakia; ^2^ Slovak Alliance for Rare Diseases, Pezinok, Slovakia; ^3^ State Institute for Drug Control, Bratislava, Slovakia

**Keywords:** orphan medicinal product, rare diseases, data-based decision-making, legislation—EEC, patient access to medicines

## Abstract

**Objective:** Information about the access of Slovak patients to orphan medicinal products (OMPs) in the literature is rather scarce. The main aim of the study was to analyze the accessibility and availability of OMPs to Slovak patients in the years 2010–2019.

**Methods:** The analyzed OMPs were strictly defined according to the European definition. The date of marketing authorization together with its first appearance in the positive drug list was used to count the time to reach the national market. The data from the National Health Information Centre, the Ministry of Health, and health insurance companies were used as data sources of drug usage, expenditure, consumption, reimbursement of OMPs, as well as the total number of treated patients.

**Results:** Out of the 167 OMPs on the European market, we identified 52% (87) OMPs which had any kind of costs recorded in Slovakia. Out of them, 62% (54) OMPs were directly present on the positive drug list. The remaining 33 OMPs were available on exception. The trend in accessibility and availability of OMPs in Slovakia between the years 2010 and 2019 was decreasing (57% OMPs in 2010 vs. 47% OMPs in 2019). The average time for an orphan medicinal product to reach the Slovak market was almost 4 years, 43.5 months [6—202 months]. Together, 10.4% (8 815 patients) out of the theoretical patients’ estimation according to the prevalence in the orphan designation were treated with OMPs available in Slovakia.

**Conclusion:** Presented data clearly show insufficient accessibility and availability of OMPs in Slovakia. Importance of clearly defined criteria for OMPs supporting patients and healthcare professionals’ involvement in the final decision together with other measures such as social impact, improvement of patients’ quality of life, society wide meaning, or no alternative treatment in the final decision is crucial for transparent and sustainable access to OMPs and innovative treatments in Slovakia.

## Introduction

The Slovak Republic (SR) is a small Central European country, with a total number of inhabitants of about 5.5 million. As described by Kapalla et al., 2010, Slovakia’s health insurance system is neither Bismarck nor Beveridge nor the National Health Insurance model, although it has certain features of all (Kapalla et al., 2010). Health insurance in Slovakia is based on a solidarity system that is represented by all citizens paying so-called health contributions, which are compulsory. The care and services the patients receive are independent of the amount of their contribution to the common healthcare fund. In theory, the solidarity is unlimited; however, in the real world setting, rare disease patients face serious problems accessing the available treatments. Most of the health funding (80%) is publicly financed. The sources of the revenue are mainly wage dependent; however, one-third comes from general tax and revenues to pay for contributions for some subsidized categories such as children, unemployed, students, and pensioners. Although spending on health care has increased in the past decade, healthcare expenditure as a share of gross domestic product (GDP) remained stable and much lower than the average in the European Union (EU) (6.7 *vs*. 9.8%). One-third of the current healthcare expenditure in Slovakia was allocated for pharmaceuticals and medical devices (OECD, 2019). The high share of pharmaceuticals and medical devices in Slovakia can be attributed to a small healthcare budget and small prices per service, visible for example in the low use of cross-border directive (Malinowski et al., 2019). Slovak patients cannot afford to pay the difference in costs between the services in Slovakia and most European countries. Thus, they must use an alternative way to get access to health care abroad, which is an administrative burden and time-consuming for all stakeholders, although it is covered by the health fund. This negatively impacts Slovak patients, especially rare disease patients who lack many services for their rare disease at the national level (Wilson et al., 2020).

However, high expenditure for pharmaceuticals and medical devices is one of the motivations for regulators to focus on the regulation about pricing and reimbursement of medicinal products in Slovakia. The current system was strongly affected by a healthcare reform and the new pricing and reimbursement act in 2011. Access to treatment for rare disease patients, but other patients as well, has also been negatively impacted by two novelizations of the pricing and reimbursement act performed in 2018 and 2019 (MZ SR, 2011). Since OMPs are associated with higher prices, Slovakia, similar to most Central and Eastern European countries (CEE), set up a formal health technology assessment process, and its positive decisions are associated with reimbursement (Bucek Psenkova et al., 2017, Kos, 2019; Stawowczyk et al., 2019; Malinowski et al., 2020). Differently to other CEE countries, the reimbursement decision in Slovakia is based on the cost-effectiveness threshold expressed as a multiple of the average salary with a possibility of reimbursement approval if a very strict condition regarding the number of patients in Slovakia is fulfilled (less than 1:50,000 inhabitants according to disease prevalence or authorized therapeutic indication).

The current legislative framework together with low access to treatment indicates that the whole sector could be better regulated, as concluded by the Organization for Economic Co-operation and Development (OECD, 2019).

Currently, Slovakia is preparing the third novelization of the pricing and reimbursement act. This was one of the motivations behind this study to produce analysis with more complex data, focusing attention on rare disease patients and their access to treatments.

The main aim of this study is to analyze the access of Slovak patients to orphan medicinal products (OMPs), in the years 2010–2019 by analyzing financial reimbursement from publicly available databases. Patients’ data come from insurance companies. There is no published article with such complex data on access to OMPs for Slovak patients to date; most of the articles refer rather to the process of drug reimbursement (Kawalec et al., 2017; Malinowski et al., 2019; Tesar et al., 2019; Németh et al., 2020) or access to a limited number of OMPs (Blankart et al., 2011; Tesar et al., 2017).

## Methods

Slovakia uses reference pricing; thus, it has the third-cheapest drugs in the European Union. Slovak patients can access OMPs in two ways:

1) Directly, if the drug is on the positive drug list,
2) On exception, without guaranteed access, where approval of reimbursed treatment with the drug is provided to the physician on the patients’ basis.

Direct access means the medicine is on the positive drug list. The positive drug list is the reimbursement list, which specifies medicines according to total or partial reimbursement. It is updated on a monthly basis and freely available on the website of the Ministry of Health. OMPs are mainly fully covered. The drug is added to the positive drug list at the request of the marketing authorization holder. It must fulfill certain criteria, none of which, however, take the orphan status into account. Since 2011, the cost-effectiveness threshold was implemented directly in the pricing and reimbursement act. Since 2018, the formal health technology assessment (HTA) is performed by the expert working group on pharmacoeconomics, clinical outcomes, and health technology assessment at the Ministry of Health. It focuses on cost-effectiveness and budget impact. The final opinion may include clinical outcomes or health technology assessments. The OMP status is considered minimally. Medicines receive their orphan status with just 2 out of 6 points in multi-criteria decision analysis (MCDA). The base to pass is 35 points. Additional points from the MCDA can be added or subtracted. The total score in the interval of 28–41 points is multiplied by the average monthly wage valid two years ago to derive the cost-effectiveness threshold. However, medicines indicated for diseases with prevalence less than 1:50,000 do not need to provide a pharmacoeconomic assessment.

Exceptional access means the drug is reimbursed for a certain patient or patient group. This decision is a subject of the health insurance company and is mainly dependent on negotiations with the manufacturer. There is no special budget dedicated for exceptional reimbursement; it is a part of the budget for all medicines. There are no clear criteria for exceptional reimbursement. Neither the orphan status, medical need, or existing alternative treatment is considered.

In this study, we strictly used the definition of the OMP according to the European Commission definition (Regulation EC, 2000). The list of drugs with valid orphan status comes from the European Medicines Agency (EMA)—supplementary file (Agendas, 2018). The OMP should have at least one valid orphan designation (OD) and marketing authorization as the OMP for treatment of rare condition/conditions between Jan 1, 2010, and Dec 31, 2019. For every OMP, the time between EMA approval and OD expiration, withdrawal, or approval surrender has been established. All data collected and described below were studied for the lifetime interval of a particular OMP. Together, 167 OMPs fit this criterion.

The data about access to OMPs in Slovakia were acquired mainly from publicly available data sources at the national level. The full list of OMPs reimbursed in Slovakia and their consumption comes from the National Health Information Centre database. The list of directly available OMPs for Slovak patients comes from the positive drug list published on a monthly basis on the website of the Slovak Ministry of Health. Drugs that were present in the database of the National Health Information Centre but were missing in the positive drug list have been marked as drugs reimbursed on exception.

The time to reach the Slovak market was counted as the time between the EMA marketing authorization date and the date of the first appearance of the OMP in the positive drug list. The data about the total number of patients treated with a particular OMP/year, as well as the total number of unique patients treated with a certain OMP, come directly from payers–health insurance companies. All three insurance companies kindly provided the data about insured patients treated with every OMP.

The data about prevalence at the time of marketing authorization come from the OD. This number was used to count the theoretical estimation of the total number of patients in Slovakia with a certain rare disease. Out of the theoretical estimation of the total number of patients in Slovakia, the percentage of really treated patients with a certain disease was counted.

## Results

### Access to OMPs: Europe *vs*. Slovakia

In ten years (2010—2019), Slovak patients were treated with 52% (87) of the available OMPs. Out of them, 21 OMPs were on the positive drug list before the changes of the reimbursement act in 2011; 34 other OMPs were added to the positive drug list in the period 2011—2019. The remaining 32 OMPs were provided on exception.

**FIGURE 1:**
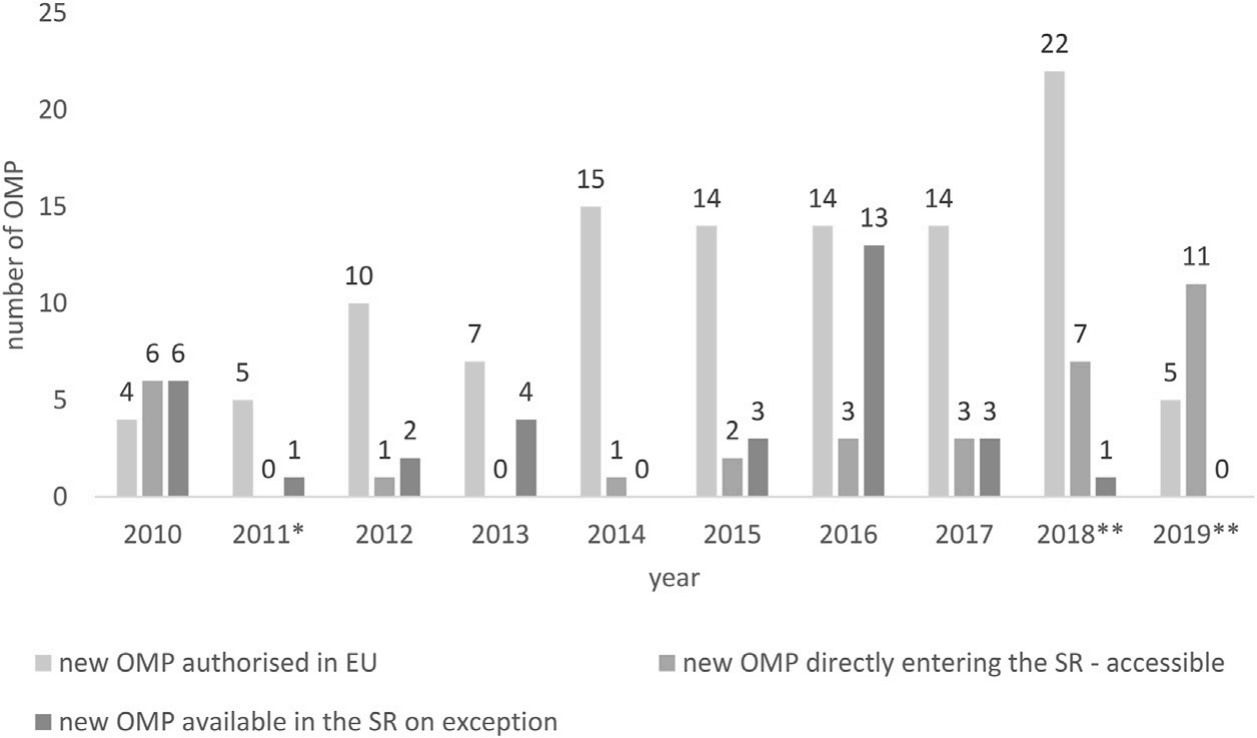
New OMPs authorized in the European Union *vs*. new OMPs accessible/available in the Slovak Republic. * New pricing and reimbursement Act, ** Novelization of the pricing and reimbursement Act.

Since the healthcare reform in 2011, the annual growth of new OMPs to Slovak patients was rather low (Figure 1). Meanwhile, during the years 2010–2019, every year on average, 11.3 [4–22] OMPs were authorized in Europe annually; the average of new OMPs accepted in the positive drug list in Slovakia every year was less than half of the European average 4.3 [0 -11]. If we consider the availability on exception, on average, another 3.3 OMPs per year [0—13] were available for Slovak patients. However, there were years, such as in 2019, when no OMP was provided on exception.

### OMPs Missing on the Slovak Market

**FIGURE 2:**
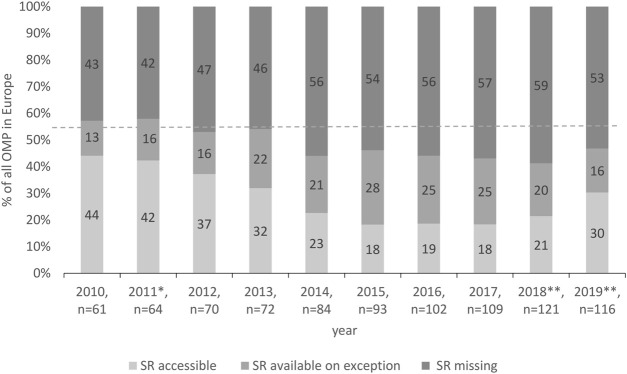
Access to OMPs in Slovakia. * New pricing and reimbursement Act, ** Novelization of the pricing and reimbursement Act.

Further detailed analysis of the availability of OMPs in Slovakia identified an increasing number of missing OMPs on the national market in Slovakia, 43% in 2010 *vs*. 53% in 2019 (Figure 2). Still, every second OMP available in Europe was missing in Slovakia. In ten years, together, 87 OMPs were used in Slovakia. Considering the access and using the terminology of Pejcic et al. (2018), 62% of them have been accessible for any patient with a given indication in the ten-year period. The remaining 38% of OMPs were available on exception.

### OMPs According to ATC Classification

**FIGURE 3:**
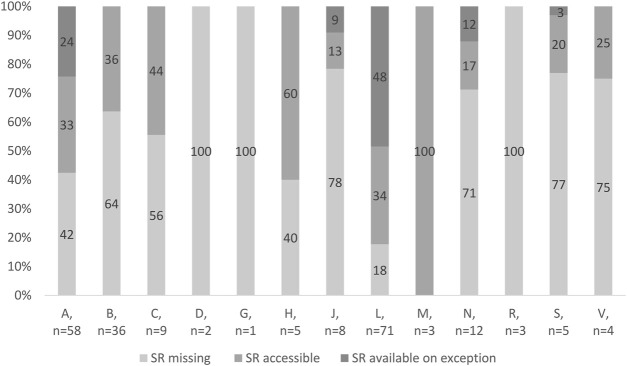
Access to OMPs in Slovakia.

Figure 3 shows access to OMPs in Slovakia according to anatomic-therapeutic classes (ATC classes). In ATC classes D (dermatological), G (genitourinary system and sex hormones), and R (respiratory system), no OMP is accessible in the SR. On the other hand, all OMPs indicated for rare musculoskeletal diseases are accessible in the SR. Only 18% of OMPs indicated for rare oncologic diseases are absolutely missing. However, almost half of the antineoplastic and immunomodulating agents are provided on exception. In the ATC class A (alimentary system), almost every second OMP indicated for a rare metabolic disease is missing. However, rare metabolic diseases in Slovakia are treated with accessible OMPs rather than available OMPs.

### Time to Reach the Slovak Market

**FIGURE 4:**
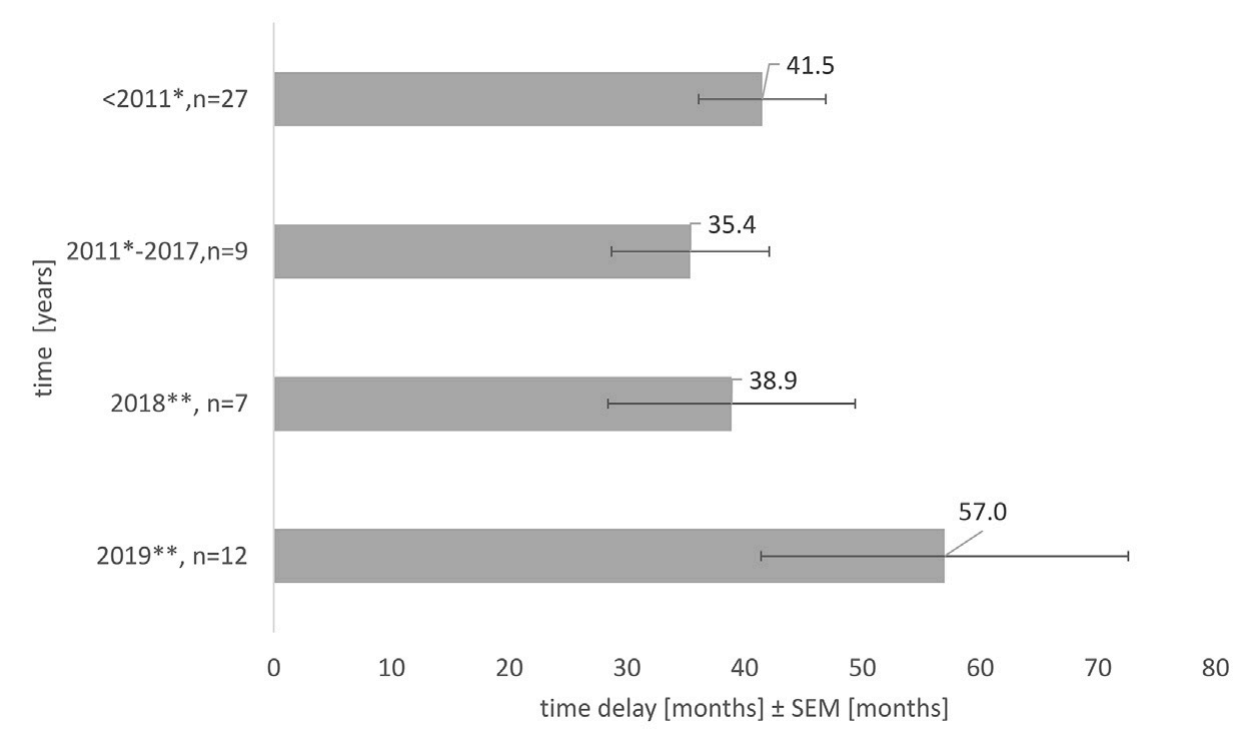
Average time delay in access to OMPs in Slovakia, the impact of the health reform, and novelization of the pricing and reimbursement Act, SEM = standard error of the mean, * New pricing and reimbursement Act, ** Novelization of the pricing and reimbursement Act.

In the 10 years reviewed, the delay to reach the Slovak market was on average almost 4 years, 43.5 months [6—202 months]. Figure 4 depicts an average delay in OMPs accessible for every patient. It stresses the fact that the medicines which reached the market due to the reimbursement novelization in 2018, were those that were authorized in the EU 3 years—38.9 ± 10.5 months earlier, respectively, 5 years earlier, 57.0 ± 15.6 months earlier in 2019. Considering another fact, there exists an exceptional reimbursement of OMPs in Slovakia; more than half (57.1%) of the OMPs which reached the Slovak market in 2018 were available on an individual basis before, respectively 58.3% of the OMPs in 2019.

### Expenditure for OMPs

An overall analysis of expenditure for OMPs during the years 2010–2019 shows an increase (Table 1). The detailed comparison of costs of accessible and available exceptionally reimbursed OMPs indicates an extensive increase of drug expenditure for the treatment of patients with rare diseases in the exceptional regime. As seen in our data, there is no relationship between the expenditure for OMPs given on exception and directly accessible OMPs on the positive drug list. However, as seen in Table 1, the increase of expenditure for OMPs from the total expenditure for medicines in Slovakia was rather gradual, whereas the increase of expenditure for OMPs on exception was unpredictable.

**TABLE 1:** Trends in expenditure for OMPs and cumulative number of rare disease patients treated with OMPs per year.

Year	% For OMPs from expenditure for all medicines	% Of exceptionally reimbursed OMPs from expenditure for all OMPs	Cumulative number of patients treated with accessible OMPs	Cumulative number of patients treated with exceptionally reimbursed OMPs	Cumulative number of patients treated with OMPs
2010	2.8	2.2	1,405	44	1,449
2011*	3.4	4.7	1,739	72	1,811
2012	3.7	8.1	1,781	58	1,832
2013	3.2	17.2	1,375	108	1,483
2014	3.6	17.2	1,527	152	1,679
2015	3.7	23.4	1,552	231	1,783
2016	4.2	27.0	1,583	295	1,878
2017	4.2	20.8	1,635	309	1,944
2018**	4.7	15.0	1,873	309	2,182
2019**	5.3	14.2	1,926	271	2,197

* New pricing and reimbursement Act, ** Novelization of the pricing and reimbursement Act.

### Patients Treated With OMPs

The data set described above refers to 8,815 individual patients treated with OMPs in Slovakia in 10 years. In the 10 years, the total number of patients treated with OMPs increased by 52% compared to the baseline.

The average number of patients treated with OMPs per year was 1,825 pts [1,449–2,197], and the trend was increasing, with a deep decrease in 2013 and a very mild increase in 2019. Table 1 represents the cumulative number of patients treated with OMPs per year, considering the way of access. The proportion of patients treated with exceptionally reimbursed OMPs varied from 3.1% in 2010 to 18.9% in 2017.

### Prevalence at the Time of Orphan Designation vs. Real Number of Treated Patients

**FIGURE 5:**
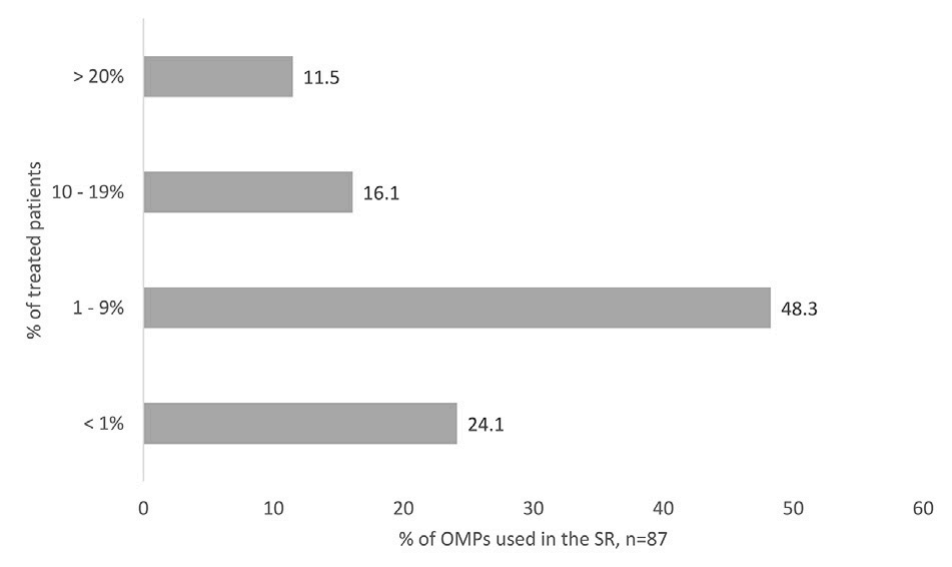
OMPs accessed in the SR, classified according to the percentage of treated patients. The Percentage is counted out of the patients’ number theoretically estimated from the prevalence in orphan designation at the time of marketing authorization.

The real number of treated patients (8,815) is markedly lower than the theoretical estimation according to the prevalence in OD at the time of marketing authorization (84,399 patients). Together, treated patients in Slovakia account for 10.4% of the theoretical estimation according to the prevalence in OD at the time of marketing authorization. There were just 11.5% OMPs that were used in more than 20% of rare disease patients according to theoretically estimated patients’ number in Slovakia. Most of the OMPs (40%) were indicated for oncologic diseases. The majority of the OMPs in Slovakia (48.3%) were used in 1–9% of the patients out of the theoretically estimated total number of patients with a certain rare disease (Figure 5).

## Discussion

Differences in access to OMPs has been an ongoing problem since orphan drug regulation was introduced (Parisse-Brassens, 2009; Tejada, 2011). The data from Slovakia are usually absent in different international comparisons analyzing access to OMPs. Importantly, several methods or subsets of analyzed OMPs are used, thus hindering direct comparisons. For example, medicines that were already without orphan status at the time of analysis are counted as OMPs (Detiček et al., 2018). Although using this approach overestimates the real number of available OMPs on the national market, it still successfully identifies the differences in access between countries. Germany and France are identified as countries with a higher number of OMPs in comparison to Central European countries such as Slovakia and Poland, where a substantial space for improvement is present (Detiček et al., 2018; Stawowczyk et al., 2019; Zamora et al., 2019; Czech et al., 2020). Nevertheless, in our study we analyzed OMPs only with valid OD. According to our results in the past years (2010–2019), every other OMP in Slovakia was missing, and the trend was negative. This is in line with the published data about low access to OMPs in other Central and Eastern European countries (Caban et al., 2016; Logviss et al., 2016) as well as Balkan and Eurasian countries (Iskrov et al., 2012; Pejcic et al., 2018; Czech et al., 2020). On the other hand, it is in deep contrast with Germany, France, and the Netherlands (Orofino et al., 2010; Bourdoncle et al., 2019; Czech et al., 2020), where access to OMPs is much better. The delay in access to OMPs in Slovakia is extremely long if compared with other countries (Detiček et al., 2018). OMPs are launched in Slovakia on average 3 or even 5 years after the drug has been authorized. None of the novelizations managed to change the situation and support timely access of OMPs to the Slovak patients. Drugs that were added to the positive drug list in 2018, thanks to the long-expected reimbursement novelization, were mainly drugs that were used in exceptional regimes before. Thus, the novelization in 2018 did not bring new innovative treatment options to rare disease patients or healthcare professionals. It just managed the uncertainty of access and the administrative burden of the healthcare professionals when treating with exceptionally reimbursed OMPs.

Factors that influence the delay of OMPs when coming to the national market are the population size, total healthcare expenditure per capita, rare disease policy, and level of expertise, as well as pricing and power in reimbursement, negotiations, and the company’s decision to delay the OMP in the country due to reference pricing. From this point of view, Slovakia is a post-socialistic country with lower healthcare expenditure per capita than the European average, lacking experience in rare disease policy and expertise (Kanavos et al., 2017; Czech et al., 2020). A further obstacle is the uniform reference pricing as Slovakia has the third cheapest drugs in the European Union. The OMP status is considered minimally. As described by some authors, external reference pricing has perverse consequences when country-specific economic parameters are considered (Young et al., 2017). This is also the case in Slovakia. Since Slovakia was missing in their analysis, we searched for countries with a similar GDP spent on health care according to the OECD and at the same time countries involved in the analysis of (Young et al., 2017; OECD, 2019). These were Greece and Poland. We identified that OMPs in Slovakia are between 2.95 times [1.45–4.65] (Greece) and 5.35 times [4.21–7.37] (Poland) more costly than those of the United Kingdom. On the other side, the budget impact of OMPs in Slovakia in 2019 was 5.3%, which fits with the estimation by Schey (Schey et al., 2011).

Pricing and power in reimbursement and negotiations in Slovakia are poor. Although Slovakia signed together with other Visegrád countries (Poland, Czech Republic, and Hungary), Croatia, and Lithuania, the memorandum of understanding of fair cooperation in the area of fair pricing for medicines does not take advantage of it (Annemans et al., 2017; Ministry of Foreign Affairs of the Republic of Poland, 2017). This approach seems to be more accepted by larger markets, but as seen in the example of BeNeLuxA, smaller countries can take the advantage of it as well (Michalopoulos, 2018). Of further concern is the fact that high costs for OMP are compensated by flat expenditure for non-OMPs and increased volumes of cheaper generics/biosimilars and developments toward more specialized targeting of diseases (Mestre-Ferrandiz et al., 2019). Clearly defined and transparent criteria for managed entry agreements (MEAs) respecting good practices in MEAs, respectively general procurement at the national level or even the international level, are one of the needed approaches also supported by the European Commission (Pauwels et al., 2017; Bucics, 2020). The alternative is patient supply on exception. However, this is highly unpredictable. As seen from our results in 2016, patients access 13 new OMPs *via* this route, whereas in 2019, no new OMP was reimbursed on an exceptional basis. This might be explainable by novelization in 2019 as well as the higher number of OMPs which were regularly launched on the Slovak market. On the other hand, exceptional reimbursement is not guaranteed in Slovakia, even if the medicine is the only therapeutic option for a patient, or there is an unmet medical need, respectively, if it is end-of-life medicine. No use of exceptional reimbursement of OMPs absolutely blocks access to medicines for patients who are in direct instead of dire need and need fast and flexible solutions. In this context, it is worth mentioning that in Slovakia, all medicines are paid from the same fund independently of the way of access. This is different from other countries such as the UK, Belgium but also Poland, Hungary, or the Czech Republic, where special funds exist for medicines that were not reviewed by the authorities or were not accepted or are financed *via* hospital extra budgets (the Netherlands) or are considering other criteria as the end-of-life criterion (Morel et al., 2013; Ferrario and Kanavos, 2015). Since expenditure for medicines in Slovakia was gradually increasing, together with the total expenditure for OMPs, ceasing the exceptional access to OMPs was the easiest option to lower the expenditure for medicines in a short time. However, this approach does not solve the budgetary uncertainty associated with OMPs in long term. On the contrary, it limits patients’ access to OMPs, negatively impacts healthcare professional motivation and involvement, and stresses the importance of clear criterion benchmarks and flexibility for exceptional reimbursement (Lagarde et al., 2019). It is also in deep contrast with the rare disease health policy in Slovakia (MZ SR, 2012; Slovak Alliance for Rare diseases, 2019; Hedley et al., 2021; MZ SR, 2021). On the other hand, it partially explains the late involvement of Slovakia in European reference networks. Slovakia is involved in seven European reference networks (ERNs) as an affiliated partner and in four ERNs as a full member (European Commission, 2021).

Apart from financial uncertainty, there exist other concerns, including bad estimation of the total number of rare disease patients and uncontrolled growth of the total number of treated patients. As presented in our results, these are rather unfounded in the case of Slovakia. Almost 9 in 10 OMPs available in Slovakia were used in less than 20% of patients estimated according to the prevalence in OD at the time of marketing authorization. It must also be considered that transparent tools exist for managing this kind of uncertainty such as cap fixation, outcome guarantee, coverage with evidence, access schemes, limiting prescribing to those subgroups who are most likely to benefit, use of biomarkers, or physician’s certification.

The European Commission is supporting the generation of more precise numbers about rare disease patients at the European level by creating European registries for rare diseases involving European reference networks (Hedley et al., 2018). Since 2016, Slovakia has been generating its rare disease registry. At the end of 2018, there were 6,071 laboratory-approved case studies of 500 different rare diseases. However, this registry includes a few patients with rare diseases treatable with OMPs. One reason for this might be that the registry is primarily run by the genetic society, mainly concentrated on genetic inborn errors, developmental disorders, and birth defects. Nevertheless, this imperfection is easily manageable even by the prepared novelization. One option might be that the condition to reimburse OMPs for rare disease patients is possible only in case the patient data are involved in the national rare disease registry. Another relatively easily manageable option is to connect the rare disease registry with other registries and newborn screening data. However, all of them might be just supportive of the most important action - to support the implementation of Slovak rare disease patients’ data in European registries. As seen in metabolic diseases, this is not the case, and data from Slovakia are missing in this analysis (MetabERN collaboration group et al., 2020). Although the current reimbursement legislation does not pay special attention to the creation of high-quality data sources, there is no doubt it is justified (De Jongh et al., 2021; Gutierrez et al., 2015).

Finally, a relatively low number of rare disease patients received treatment with OMPs. Only 10.4% of the rare disease patients received treatment out of the theoretical estimation according to the prevalence in OD. In the10-year period, the total number of patients treated with OMPs in Slovakia increased by 52% compared to the baseline (1,449 pts in 2010 *vs*. 2,197 in 2019). However, the total number of OMPs increased by 90% compared to the baseline. The expenditure for OMPs was steeply increasing; unfortunately, this is not the case for the total number of rare disease patients, who received treatment with OMPs. In 10 years, the number of rare disease patients treated with OMPs increased by 52%; however, the expenditure increased by 140%. No clearly formulated criteria for OMPs, including managed entry agreements, result in preferably negative decisions or no interest of the marketing authorization holder to launch the product in Slovakia compared to other countries, which look for innovative reimbursement approaches to provide the treatments for their population (Ferrario and Kanavos, 2013; Morel et al., 2013; EXPH (EXpert Panel on effective ways of investing in Health), 2018; Michelsen et al., 2020; Blonda et al., 2021).

Uncertainties will always exist and may also evolve. Some may be unavoidable at an early stage but may be addressed later. In Slovakia, the main issues seem to be a lack of mutual understanding between regulators, payers, and the pharmaceutical industry together with insufficient high-quality data sources to support evidence-based decisions and courage for innovative reimbursement approaches. Innovative reimbursement approaches need fully developed legislation with a sufficient space for discussions and data gathering. Of note are societal preferences. In this case, the Slovak population is supporting the weak ones. The solidarity of the Slovak society is evidenced by the treatment of spinal muscular atrophy (SMA). In an extremely short time, Slovak citizens crowdfunded for a single-dose adeno-associated viral vector gene therapy (Čiefová, 2020). Another example is the continuing strong support of the Slovak patients’ group for cystic fibrosis to the cystic fibrosis community in Ukraine (V4 Future CF, 2021). However, it needs to be translated into the willingness of politicians and into the legislative documents as the prepared novel of the reimbursement act.

## Conclusion

The trend in accessibility and availability of OMPs in Slovakia between 2010 and 2019 was decreasing. In 2019, in Slovakia, every second OMP was missing. The implementation of the cost-effectiveness threshold directly in the pricing and reimbursement act created serious obstacles for OMPs to enter the Slovak market. None of the novelizations of the reimbursement act (2018, 2019) managed to solve the problem of insufficient access to OMPs. The average time for an OMP to reach the Slovak market was prolonged, reaching 4.8 years’ delay in 2019. OMPs were preferably directly accessible. The proportion of expenditure for OMPs out of the expenditure for all medicines increased by 53% in 10 years. In 10 years, the number of rare disease patients treated with OMP increased by 52%; however, the expenditure increased by 140%, mainly due to the extensive and unpredictable increase of expenditure for OMPs reimbursed in the exceptional regime. Only 10.4% of patients of the theoretical estimation according to the prevalence in OD at the time of marketing authorization were treated with OMPs in Slovakia.

The small drug market in Slovakia is even smaller when considering the low prevalence of rare diseases. Of note are further factors such as low GDP, insufficient and complicated data gathering, time and money complicated diagnostics, single-source financing, political rather than data-based decision-making, and legislation imperfections. On the other hand, a small population country is more manageable in case of precise data gathering and their interconnections and monitoring. High-quality data are necessary to form decisions based on data. The novel reimbursement legislation, which is in preparation in Slovakia now in 2021, creates a unique opportunity to do so and to adopt good practices from different European countries to support innovative treatments not only for rare disease patients but also to increase the quality of care for all citizens with innovative treatments.

## Limitations

There are several limitations to our work. The first of them is the accuracy of the data regarding the expenditure. It does not reflect discounts provided by the marketing authorization holder; thus, the real expenditure for OMPs is overestimated. On the other hand, the discount could be provided on the regularly accessible OMPs and on the ones available on exception; thus, the proportionality of the data is relatively precise. Of note is also the fact that this data source is used in all statistics Slovakia provides and uses for further analyzing and decision-making. Moreover, a publicly available source of more precise financial data is missing. The second limitation is missing information about MEA. Since the information about MEA or the type of MEA is not publicly available, we did not have the opportunity to identify the type of MEA nor had the opportunity to identify the weaknesses of MEA.

A further limitation is in the total number of treated patients. Importantly, most of the OMPs in our analysis were not used for the whole 10 years; thus, it is challenging to identify the prevalence of patients with a certain rare disease as well as the total number of rare disease patients indicated for treatment with a certain OMP in Slovakia. The real numbers of rare disease patients in Slovakia were much lower than estimations according to the prevalence in OD, stressing the need for improvement in better diagnostics.

## Data Availability

The original contributions presented in the study are included in the article/Supplementary Material, further inquiries can be directed to the corresponding author.
