# Supplementary material for: Availability and Accessibility of Orphan Medicinal Products to Patients in Slovakia in the Years 2010–2019
Source: Front Pharmacol. 2022 Jan 26;13:768325. doi: 10.3389/fphar.2022.768325 (PMC8826087; doi:10.3389/fphar.2022.768325)
Supplement: Supplementary file 2 [file DataSheet1.PDF]

MAA Product Number	MA Authorisation Type (prod level)	Accelerated Review	MAA/MAH	Medicinal Product	INN	ATC Code	EU Birth date	EU Birth Year	Marketing Authorisation Valid Until Date	MAA Product Status	MA withdrawn date (other table)	Withdrawn /Expired Date	Orphan Status	Prevalence (per 10,000)	Significant Benefit	Broadest Disease Group	Type of Medicinal Product	Therapeutic Indication - Summary	Market Exclusivity Planned Expiry Date	Orphan Designation Date	Orphan Designation Procedure Number	Orphan Designation Number	Designated Orphan Indication	comment
EMEA/H/C/002455	CONDITIONAL	N	Takeda Pharma A/S	Adcetris	brentuximab vedotin	L01XC12	25.10.2012	2012	30.10.2020	VALID			GRANTED			peripheral t-cell lymphoma	Monoclonal antibodies	treatment Hodgkin lymphoma (HL) and systemic anaplastic large cell lymphoma (sALCL)	29.10.2022	15.1.2009	EMA/OD/072/08	EU/3/08/595	Treatment of peripheral t-cell lymphoma	
EMEA/H/C/002737	STANDARD	N	Bayer AG	Adempas	riociguat	C02KX05	27.3.2014	2014	10.8.9999	VALID			GRANTED			pulmonary arterial hypertension including treatment of chronic thromboembolic pulmonary hypertension	Single	treatment of Chronic thromboembolic pulmonary hypertension (CTEPH) and Pulmonary arterial hypertension (PAH)	30.3.2024	20.12.2007	EMA/OD/088/07	EU/3/07/518	Treatment of pulmonary arterial hypertension including treatment of chronic thromboembolic pulmonary hypertension	
EMEA/H/C/001038	STANDARD	N	Novartis Europharm Limited	Afinitor	everolimus	L01XE10	3.8.2009	2009	10.8.9999	VALID			EXPIRED			Renal cell carcinoma	Single	treatment of advanced renal cell carcinoma and treatment of neuroendocrine tumours of pancreatic origin	4.8.2019	5.6.2007	EMA/OD/005/07	EU/3/07/449	Treatment of Renal cell carcinoma	
EMEA/H/C/000477	STANDARD	N	Genzyme Europe BV	Aldurazyme	laronidase	A16AB05	10.6.2003	2003	10.8.9999	VALID			EXPIRED			Mucopolysaccharidosis type I	Recombinant proteins	treatment of Mucopolysaccharidosis I	11.6.2013	14.2.2001	EMA/OD/042/00	EU/3/01/022	Treatment of Mucopolysaccharidosis type I	
EMEA/H/C/004258	STANDARD	N	Takeda Pharma A/S	Alofisel	darvadstrocel	L04AX08	23.3.2018	2018	27.3.2023	VALID			GRANTED			Anal fistula	Allogeneic (Cell therapy)	treatment of complex perianal fistula(s)	26.3.2028	8.10.2009	EMA/OD/054/09	EU/3/09/667	Treatment of Anal fistula	
EMEA/H/C/004142	STANDARD	N	Swedish Orphan Biovitrum AB (publ)	Alprolix	eftrenonacog alfa	B02BD04	12.5.2016	2016	13.5.2021	VALID			GRANTED			Haemophilia B	Recombinant proteins	treatment and prophylaxis of bleeding in patients with haemophilia B	12.5.2026	8.6.2007	EMA/OD/012/07	EU/3/07/453	Treatment of Haemophilia B	
EMEA/H/C/004379	STANDARD	N	Ammtek	Amglidia	glibenclamide	A10BB01	24.5.2018	2018	28.5.2023	VALID			GRANTED			neonatal diabetes	Single	treatment of neonatal diabetes	27.5.2028	15.1.2016	EMA/OD/149/15	EU/3/15/1589	Treatment of neonatal diabetes	
EMEA/H/C/001131	STANDARD	N	Novartis Europharm Limited	Arzerra	ofatumumab	L01XC10	19.4.2010	2010	28.4.2020	SURRENDERED			EXPIRED			chronic lymphocytic leukaemia	Monoclonal antibodies	treatment of chronic lymphocytic leukaemia (CLL)	20.4.2020	7.11.2008	EMA/OD/051/08	EU/3/08/581	Treatment of chronic lymphocytic leukaemia	
EMEA/H/C/000752	EXCEPTIONAL	N	Novartis Europharm Limited	Atriance	nelarabine	L01BB07	22.8.2007	2007	24.8.2022	VALID			EXPIRED			Acute lymphoblastic leukaemia	Single	treatment of T-cell acute lymphoblastic leukaemia (T-ALL) and T-cell lymphoblastic lymphoma (T-LBL)	23.8.2017	16.6.2005	EMA/OD/015/05	EU/3/05/293	Treatment of Acute lymphoblastic leukaemia	
EMEA/H/C/004338	CONDITIONAL	N	Merck Europe B.V.	Bavencio	avelumab	L01XC31	18.9.2017	2017	20.9.2020	VALID		7.10.2019	WITHDRAWN			Merkel cell carcinoma	Monoclonal antibodies	treatment of Merkel cell carcinoma (MCC)		14.12.2015	EMA/OD/150/15	EU/3/15/1590	Treatment of Merkel cell carcinoma	
EMEA/H/C/004119	STANDARD	N	Pfizer Europe MA EEIG	BESPONSA	inotuzumab ozogamicin	L01XC26	29.6.2017	2017	3.7.2022	VALID			GRANTED			Acute lymphoblastic leukaemia	Biologically derived proteins and peptides	treatment B-cell precursor acute lymphoblastic leukaemia (ALL)	2.7.2027	7.6.2013	EMA/OD/194/12	EU/3/13/1127	Treatment of Acute lymphoblastic leukaemia	
EMEA/H/C/003731	STANDARD	N	Amgen Europe B.V.	BLINCYTO	blinatumomab	L01XC19	23.11.2015	2015	25.11.2023	VALID			GRANTED			Acute lymphoblastic leukaemia	Monoclonal antibodies	treatment of Philadelphia chromosome negative relapsed or refractory B-precursor acute lymphoblastic leukaemia	24.11.2025	24.7.2009	EMA/OD/029/09	EU/3/09/650	Treatment of Acute lymphoblastic leukaemia	
EMEA/H/C/002373	CONDITIONAL	N	Pfizer Europe MA EEIG	Bosulif	bosutinib	L01XE14	27.3.2013	2013	2.4.2021	VALID			EXPIRED			chronic myeloid leukaemia	Single	treatment of myelogenous leukemia	1.4.2023	4.8.2010	EMA/OD/160/09	EU/3/10/762	Treatment of chronic myeloid leukaemia	
EMEA/H/C/004065	EXCEPTIONAL	Y	BioMarin International Limited	Brineura	cerliponase alfa	A16AB17	30.5.2017	2017	1.6.2022	VALID			GRANTED			neuronal ceroid lipofuscinosis type 2	Recombinant proteins	treatment of neuronal ceroid lipofuscinosis type 2	31.5.2027	12.3.2013	EMA/OD/177/12	EU/3/13/1118	Treatment of neuronal ceroid lipofuscinosis type 2	
EMEA/H/C/001252	STANDARD	N	Pharmaxis Europe Limited	Bronchitol	mannitol	R05CB16	13.4.2012	2012	10.8.9999	VALID			GRANTED	1.30	Y	Cystic fibrosis	Single	treatment of cystic fibrosis (CF)	17.4.2022	7.11.2005	EMEA/OD/062/05	EU/3/05/325	Treatment of cystic fibrosis	
EMEA/H/C/000472	STANDARD	N	Pierre Fabre Medicament	Busilvex	busulfan	L01AB01	9.7.2003	2003	10.8.9999	VALID		11.7.2013	EXPIRED		Y	Conditioning treatment prior to haematopoietic progenitor cell transplantation	Single	treatment prior to haematopoietic progenitor cell transplantation		29.12.2000	EMA/OD/024/00	EU/3/00/011	Conditioning treatment prior to haematopoietic progenitor cell transplantation	
EMEA/H/C/004426	STANDARD	N	Ablynx NV	Cablivi	caplacizumab	B01AX07	31.8.2018	2018	4.9.2023	VALID			GRANTED			Thrombocytopenic purpura	Monoclonal antibodies	indicated for the treatment of acquired thrombotic thrombocytopenic purpura (aTTP).	3.9.2028	30.4.2009	EMA/OD/109/08	EU/3/09/629	Treatment of Thrombocytopenic purpura	
EMEA/H/C/000461	STANDARD	N	Recordati Rare Diseases	Carbaglu	carglumic acid	A16AA05	24.1.2003	2003	10.8.9999	VALID			EXPIRED			N-acetylglutamate synthetase (NAGS) deficiency	Single	treatment of hyperammoniemia due to isovaleric acidaemia, methylmalonic acidaemia and propionic acidaemia	27.1.2013	18.10.2000	EMA/OD/002/00	EU/3/00/007	Treatment of N-acetylglutamate synthetase (NAGS) deficiency	
EMEA/H/C/000996	STANDARD	N	Gilead Sciences Ireland UC	Cayston	aztreonam	J01DF01	21.9.2009	2009	10.8.9999	VALID			GRANTED			gram negative bacterial lung infection in cystic fibrosis	Single	The suppressive therapy of chronic pulmonary infections due to Pseudomonas aeruginosa in patients with cystic fibrosis (CF) aged 18 years and older	22.9.2019	21.6.2004	EMA/OD/006/04	EU/3/04/204	Treatment of gram negative bacterial lung infection in cystic fibrosis	
EMEA/H/C/000796	STANDARD	N	Noventia Pharma Srl	Ceplene	histamine dihydrochloride	L03AX14	7.10.2008	2008	10.8.9999	VALID			EXPIRED			acute myeloid leukaemia (AML)	Single	treatment of myeloid leukaemia	8.10.2018	11.4.2005	EMA/OD/094/04	EU/3/05/272	Treatment of acute myeloid leukaemia (AML)	
EMEA/H/C/003724	STANDARD	N	Genzyme Europe BV	Cerdelga	eliglustat	A16AX10	19.1.2015	2015	10.8.9999	VALID			GRANTED			Gaucher disease	Single	treatment of Gaucher disease type 1	20.1.2025	4.12.2007	EMA/OD/066/07	EU/3/07/514	Treatment of Gaucher disease	
EMEA/H/C/003855	STANDARD	Y	BPL Bioproducts Laboratory GmbH	Coagadex	human coagulation factor X	B02BD13	16.3.2016	2016	18.3.2021	VALID			GRANTED			hereditary factor X deficiency	Extracted proteins	treatment of factor X deficiency	17.3.2026	17.9.2007	EMA/OD/044/07	EU/3/07/471	Treatment of hereditary factor X deficiency	
EMEA/H/C/002640	CONDITIONAL	N	Ipsen Pharma	Cometriq	cabozantinib	L01XE26	21.3.2014	2014	26.3.2021	VALID			GRANTED			medullary thyroid carcinoma	Single	treatment of medullary thyroid carcinoma	25.3.2024	6.2.2009	EMA/OD/088/08	EU/3/08/610	Treatment of medullary thyroid carcinoma	
EMEA/H/C/002734	STANDARD	N	Basilea Pharmaceutica Deutschland GmbH	Cresemba	isavuconazole	J02AC05	15.10.2015	2015	19.10.2020	VALID			GRANTED	0.06	Y	mucormycosis	Single	treatment of aspergillosis and mucormycosis	18.10.2025	4.6.2014	EMA/OD/010/14	EU/3/14/1276	Treatment of mucormycosis	
EMEA/H/C/004275	CONDITIONAL	N	Kyowa Kirin Holdings B.V.	CRYSVITA	burosumab	M05BX05	19.2.2018	2018	21.2.2021	VALID			GRANTED			X-linked hypophosphataemia	Monoclonal antibodies	treatment of X-linked hypophosphataemia (XLH)	20.2.2028	15.10.2014	EMA/OD/133/14	EU/3/14/1351	Treatment of X-linked hypophosphataemia	
EMEA/H/C/002829	STANDARD	N	Eli Lilly Nederland B.V.	Cyramza	ramucirumab	L01XC21	19.12.2014	2014	10.8.9999	VALID			EXPIRED			Gastric cancer	Monoclonal antibodies	treatment of gastric cancer	22.12.2024	4.7.2012	EMA/OD/030/12	EU/3/12/1004	Treatment of Gastric cancer	
EMEA/H/C/000678	STANDARD	N	Recordati Rare Diseases	Cystadane	betaine anhydrous	A16AA06	15.2.2007	2007	10.8.9999	VALID			EXPIRED			Homocystinuria	Single	treatment of homocystinuria	18.2.2017	9.7.2001	EMA/OD/003/00	EU/3/01/045	Treatment of Homocystinuria	
EMEA/H/C/003769	STANDARD	N	Recordati Rare Diseases	Cystadrops	mercaptamine	S01XA21	19.1.2017	2017	23.1.2022	VALID			GRANTED			Cystinosis	Single	treatment of cystinosis	22.1.2027	7.11.2008	EMA/OD/036/08	EU/3/08/578	Treatment of Cystinosis	
EMEA/H/C/002221	STANDARD	N	Janssen-Cilag International N.V.	Dacogen	decitabine	L01BC08	20.9.2012	2012	10.8.9999	VALID			GRANTED			Acute myeloid leukaemia	Single	treatment of acute myeloid leukaemia (AML)	23.9.2022	8.6.2006	EMA/OD/004/06	EU/3/06/370	Treatment of Acute myeloid leukaemia	
EMEA/H/C/004077	STANDARD	Y	Janssen-Cilag International NV	Darzalex	daratumumab	L01XC24	20.5.2016	2016	3.5.2022	VALID			GRANTED			plasma cell myeloma	Monoclonal antibodies	treatment of patients with relapsed and refractory multiple myeloma treatment of patients with relapsed and refractory multiple myeloma	23.5.2026	17.7.2013	EMA/OD/038/13	EU/3/13/1153	Treatment of plasma cell myeloma	
EMEA/H/C/002393	EXCEPTIONAL	N	Gentium S.r.l.	Defitelio	defibrotide	B01AX01	18.10.2013	2013	22.10.2023	VALID			GRANTED			hepatic veno-occlusive disease	Extracted proteins	treatment of hepatic veno-occlusive disease		29.7.2004	EMA/OD/025/04	EU/3/04/211	Prevention of hepatic veno-occlusive disease	
EMEA/H/C/002552	CONDITIONAL	N	Otsuka Novel Products GmbH	Deltyba	delamanid	J04AK06	28.4.2014	2014	30.4.2021	VALID			GRANTED			tuberculosis	Single	treatment of multidrug-resistant tuberculosis (MDR-TB)	29.4.2024	1.2.2008	EMA/OD/094/07	EU/3/07/524	Treatment of tuberculosis	
EMEA/H/C/000664	STANDARD	N	BIOCODEX	Diacomit	stiripentol	N03AX17	4.1.2007	2007	10.8.9999	VALID			EXPIRED			Epilepsy	Single	treatment of myoclonic epilepsy in infancy (SMEI, Dravet's syndrome)	8.1.2017	5.12.2001	EMA/OD/030/01	EU/3/01/071	Treatment of Epilepsy	
EMEA/H/C/000700	EXCEPTIONAL	N	Shire Human Genetic Therapies AB	Elaprase	idursulfase	A16AB09	8.1.2007	2007	10.8.9999	VALID			EXPIRED			Mucopolysaccharidosis, type II (Hunter syndrome)	Recombinant proteins	treatment of Hunter syndrome (Mucopolysaccharidosis II, MPS II)	9.1.2017	11.12.2001	EMA/OD/056/01	EU/3/01/078	Treatment of Mucopolysaccharidosis, type II (Hunter syndrome)	
EMEA/H/C/004675	STANDARD	N	GW Pharma (International) B.V.	Epidyolex	cannabidiol	N03AX24	19.9.2019	2019		VALID			GRANTED			Dravet syndrome	Single	Adjunctive therapy of seizures associated with Lennox-Gastaut syndrome (LGS) or Dravet syndrome (DS)	22.9.2029	15.10.2014	EMA/OD/083/14	EU/3/14/1339	Treatment of Dravet syndrome	
EMEA/H/C/002154	STANDARD	N	Roche Registration GmbH	Esbriet	pirfenidone	L04AX05	28.2.2011	2011	10.8.9999	VALID			GRANTED			Pulmonary fibrosis	Single	treatment of Idiopathic Pulmonary Fibrosis (IPF)	1.3.2021	16.11.2004	EMA/OD/052/04	EU/3/04/241	Treatment of Pulmonary fibrosis	
EMEA/H/C/000613	EXCEPTIONAL	N	Genzyme Europe BV	Evoltra	clofarabine	L01BB06	29.5.2006	2006	10.8.9999	VALID			EXPIRED			Acute lymphoblastic leukaemia	Single	treatment of acute lymphoblastic leukaemia	30.5.2016	5.2.2002	EMA/OD/046/01	EU/3/01/082	Treatment of Acute lymphoblastic leukaemia	
EMEA/H/C/000670	STANDARD	N	Novartis Europharm Limited	EXJADE	deferasirox	V03AC03	28.8.2006	2006	10.8.9999	VALID			EXPIRED			Iron overload	Single	treatment of chronic iron overload	30.8.2016	13.3.2002	EMA/OD/061/01	EU/3/02/092	Treatment of Iron overload	
EMEA/H/C/000370	STANDARD	N	Genzyme Europe BV	Fabrazyme	agalsidase beta	A16AB04	3.8.2001	2001	10.8.9999	VALID			EXPIRED			Fabry disease	Recombinant proteins	treatment of Fabry disease	6.8.2011	8.8.2000	EMA/OD/010/00	EU/3/00/003	Treatment of Fabry disease	
EMEA/H/C/003725	STANDARD	N	Secura Bio Limited	Farydak	panobinostat	L01XX42	28.8.2015	2015	1.9.2020	VALID			GRANTED			Multiple myeloma	Single	treatment of multiple myeloma	31.8.2025	8.11.2012	EMA/OD/113/12	EU/3/12/1063	Treatment of Multiple myeloma	
EMEA/H/C/000899	STANDARD	N	Shire Pharmaceuticals Ireland Limited	Firazyr	icatibant	B06AC02	11.7.2008	2008	10.8.9999	VALID			GRANTED			Angioedema	Single	treatment of hereditary angioedema	14.7.2020	17.2.2003	EMA/OD/054/02	EU/3/03/133	Treatment of Angioedema	
EMEA/H/C/001032	EXCEPTIONAL	N	BioMarin International Limited	Firdapse	amifampridine	N07XX05	23.12.2009	2009	10.8.9999	VALID		28.12.2019	EXPIRED			Lambert-Eaton myasthenic syndrome	Single	treatment of Lambert-Eaton Myasthenic Syndrome		18.12.2002	EMA/OD/050/02	EU/3/02/124	Treatment of Lambert-Eaton myasthenic syndrome	
EMEA/H/C/004059	STANDARD	N	Amicus Therapeutics Europe Limited	Galafold	migalastat	A16AX14	26.5.2016	2016	31.5.2021	VALID			GRANTED		Y	Fabry disease	Single	treatment of patients with Fabry disease	30.5.2026	22.5.2006	EMA/OD/105/05	EU/3/06/368	Treatment of Fabry disease	
EMEA/H/C/002799	STANDARD	N	Roche Registration GmbH	Gazyvaro	obinutuzumab	L01XC15	23.7.2014	2014	10.8.9999	VALID			GRANTED			chronic lymphocytic leukemia	Monoclonal antibodies	treatment of chronic lymphocytic leukaemia (CLL) and follicular lymphoma (FL)	23.7.2024	10.10.2012	EMA/OD/102/12	EU/3/12/1054	Treatment of chronic lymphocytic leukemia	
EMEA/H/C/000744	STANDARD	N	medac Gesellschaft fur klinische Spezialpraparate mbH	Gliolan	5-aminolevulinic acid	L01XD04	7.9.2007	2007	10.8.9999	VALID			EXPIRED			Intra-operative photodynamic diagnosis of residual glioma	Single	visualisation of malignant tissue during surgery for malignant glioma	11.9.2017	13.11.2002	EMA/OD/039/02	EU/3/02/121	Intra-operative photodynamic diagnosis of residual glioma	
EMEA/H/C/000406	STANDARD	N	Novartis Europharm Limited	Glivec	imatinib	L01XE01	7.11.2001	2001	10.8.9999	VALID		12.11.2011	EXPIRED		Y	Chronic myeloid leukaemia	Single	treatment of newly diagnosed and chronic Philadelphia chromosome (bcr-abl) positive (Ph+) chronic myeloid leukaemia (CML), gastrointestinal stromal tumours (GIST), unresectable dermatofibrosarcoma protuberans (DFSP) and recurrent and/or metastatic DFSP		14.2.2001	EMA/OD/049/00	EU/3/01/021	Treatment of Chronic myeloid leukaemia	
EMEA/H/C/002145	EXCEPTIONAL	N	uniQure biopharma B.V.	Glybera	alipogene tiparvovec	C10AX10	25.10.2012	2012	29.10.2017	VALID			GRANTED			Lipoprotein lipase deficiency	Gene therapy	treatment lipoprotein lipase deficiency	28.10.2022	8.3.2004	EMA/OD/079/03	EU/3/04/194	Treatment of Lipoprotein lipase deficiency	
EMEA/H/C/002709	STANDARD	N	Eurocept International B.V.	Granupas	para-aminosalicylic acid	J04AA01	7.4.2014	2014	10.8.9999	VALID			GRANTED			tuberculosis	Single	treatment of tuberculosis	8.4.2024	17.12.2010	EMA/OD/072/10	EU/3/10/826	Treatment of tuberculosis	
EMEA/H/C/003870	STANDARD	N	Vanda Pharmaceuticals Germany GmbH	Hetlioz	tasimelteon	N05CH03	3.7.2015	2015	7.7.2020	VALID			GRANTED			non-24-hour sleep-wake disorders in blind people with no light perception	Single	treatment of Non-24-Hour Sleep-Wake Disorder (Non-24)	6.7.2025	23.2.2011	EMA/OD/102/10	EU/3/10/841	Treatment of non-24-hour sleep-wake disorders in blind people with no light perception	
EMEA/H/C/002450	STANDARD	N	Chiesi Farmaceutici S.p.A.	Holoclar	ex vivo expanded autologous human corneal epithelial cells containing stem cells	S01XA19	17.2.2015	2015	19.2.2025	VALID			GRANTED			corneal lesions, with associated corneal (limbal) stem cell deficiency, due to ocular burns	Autologous (Cell therapy)	treatment of limbal stem cell deficiency	18.2.2025	7.11.2008	EMA/OD/025/08	EU/3/08/579	Treatment of corneal lesions, with associated corneal (limbal) stem cell deficiency, due to ocular burns	
EMEA/H/C/004061	EXCEPTIONAL	N	Leadiant GmbH	Chenodeoxycholic acid Leadiant	chenodeoxycholic acid	A05AA01	10.4.2017	2017	12.4.2022	VALID			GRANTED			inborn errors of primary bile acid synthesis	Single	treatment of inborn errors of primary bile acid synthesis	11.4.2027	16.12.2014	EMA/OD/196/14	EU/3/14/1406	Treatment of inborn errors of primary bile acid synthesis	
EMEA/H/C/002695	STANDARD	Y	Incyte Biosciences Distribution B.V.	Iclusig	ponatinib	L01XE24	1.7.2013	2013	10.8.9999	VALID			GRANTED			chronic myeloid leukaemia	Single	treatment of chronic myeloid leukaemia (CML) or Philadelphia chromosome positive acute lymphoblastic leukaemia (Ph+ ALL)	2.7.2023	2.2.2010	EMA/OD/121/09	EU/3/09/716	Treatment of chronic myeloid leukaemia	
EMEA/H/C/003955	STANDARD	N	CSL Behring GmbH	IDELVION	albutrepenonacog alfa	B02BD04	11.5.2016	2016	13.5.2021	VALID			GRANTED			haemophilia B	Recombinant proteins	prophylaxis and treatment of bleeding in all patients with haemophilia B	12.5.2026	4.2.2010	EMA/OD/117/09	EU/3/09/723	Treatment of haemophilia B	
EMEA/H/C/001109	STANDARD	N	Novartis Europharm Limited	ILARIS	canakinumab	L04AC08	23.10.2009	2009	10.8.9999	VALID			EXPIRED			Cryopirin-associated periodic syndromes	Monoclonal antibodies	treatment of cryopyrin-associated periodic syndromes (CAPS), including Muckle-Wells Syndrome (MWS), Neonatal-Onset Multisystem Inflammatory Disease (NOMID) / Chronic Infantile Neurological, Cutaneous, Articular Syndrome (CINCA) and severe forms of Familial Cold Autoinflammatory Syndrome (FCAS) / Familial Cold Urticaria (FCU); Still's Disease and Gouty arthritis	26.10.2019	20.3.2007	EMA/OD/071/06	EU/3/07/439	Treatment of Cryopirin-associated periodic syndromes	
EMEA/H/C/003791	STANDARD	N	Janssen-Cilag International NV	Imbruvica	ibrutinib	L01XE27	21.10.2014	2014	10.8.9999	VALID			GRANTED			Chronic lymphocytic leukaemia	Single	treatment of mantle cell lymphoma, chronic lymphocytic leukemia, small lymphocytic lymphoma, lymphoplasmacytic lymphoma	22.10.2024	26.4.2012	EMA/OD/156/11	EU/3/12/984	Treatment of Chronic lymphocytic leukaemia	
EMEA/H/C/002682	STANDARD	N	Celgene Europe BV	Imnovid	pomalidomide	L04AX06	5.8.2013	2013	8.8.2023	VALID			GRANTED			multiple myeloma	Single	in combination with dexamethasone is indicated in the treatment of adult patients with relapsed and refractory multiple myeloma (MM)	7.8.2023	8.10.2009	EMA/OD/053/09	EU/3/09/672	Treatment of multiple myeloma	
EMEA/H/C/000704	EXCEPTIONAL	N	Ipsen Pharma	Increlex	mecasermin	H01AC03	3.8.2007	2007	10.8.9999	VALID			EXPIRED			Growth factor-1 deficiency	Monoclonal antibodies	treatment of growth failure	6.8.2017	22.5.2006	EMA/OD/104/05	EU/3/06/373	Treatment of Growth factor-1 deficiency	
EMEA/H/C/000660	STANDARD	N	Eisai GmbH	Inovelon	rufinamide	N03AF03	16.1.2007	2007	10.8.9999	VALID			EXPIRED			Lennox-Gastaut syndrome	Single	treatment of seizures associated with Lennox-Gastaut syndrome	17.1.2019	20.10.2004	EMA/OD/047/04	EU/3/04/240	Treatment of Lennox-Gastaut syndrome	
EMEA/H/C/002464	STANDARD	N	Novartis Europharm Limited	Jakavi	ruxolitinib	L01XE18	23.8.2012	2012	10.8.9999	VALID			EXPIRED			Myelofibrosis	Single	treatment of myelofibrosis	27.8.2022	7.11.2008	EMA/OD/039/08	EU/3/08/572	Treatment of Myelofibrosis	
EMEA/H/C/004655	STANDARD	Y	Dr. Falk Pharma GmbH	Jorveza	budesonide	A07EA06	8.1.2018	2018	10.1.2023	VALID			GRANTED			eosinophilic oesophagitis	Chemicals	treatment of eosinophilic esophagitis (EoE)	9.1.2028	5.8.2013	EMA/OD/078/13	EU/3/13/1181	Treatment of eosinophilic oesophagitis	
EMEA/H/C/002494	STANDARD	Y	Vertex Pharmaceuticals (Ireland) Limited	Kalydeco	ivacaftor	R07AX02	23.7.2012	2012	25.7.2022	VALID			GRANTED			cystic fibrosis	Single	treatment of cystic fibrosis (CF)	24.7.2022	8.7.2008	EMA/OD/010/08	EU/3/08/556	Treatment of cystic fibrosis	
EMEA/H/C/004004	STANDARD	Y	Alexion Europe SAS	Kanuma	sebelipase alfa	A16AB14	28.8.2015	2015	10.8.9999	VALID			GRANTED			Lysosomal acid lipase deficiency	Recombinant proteins	treatment of enzyme replacement therapy (ERT)	31.8.2025	17.12.2010	EMA/OD/104/10	EU/3/10/827	Treatment of Lysosomal acid lipase deficiency	

MAA Product Number	MA Authorisation Type (prod level)	Accelerated Review	MAA/MAH	Medicinal Product	INN	ATC Code	EU Birth date	EU Birth Year	Marketing Authorisation Valid Until Date	MAA Product Status	MA withdrawn date (other table)	Withdrawn /Expired Date	Orphan Status	Prevalence (per 10,000)	Significant Benefit	Broadest Disease Group	Type of Medicinal Product	Therapeutic Indication - Summary	Market Exclusivity Planned Expiry Date	Orphan Designation Date	Orphan Designation Procedure Number	Orphan Designation Number	Designated Orphan Indication	comment
EMEA/H/C/003906	STANDARD	Y	HRA Pharma Rare Diseases	Ketoconazole HRA	ketoconazole	J02AB02	19.11.2014	2014	10.8.9999	VALID			GRANTED			Cushing's syndrome	Single	treatment of Cushing's syndrome	20.11.2024	23.4.2012	EMA/OD/125/11	EU/3/12/965	Treatment of Cushing's syndrome	
EMEA/H/C/002081	EXCEPTIONAL	N	Retrophin Europe Ltd	Kolbam	cholic acid	A05AA03	4.4.2014	2014	24.11.2020	VALID			GRANTED			Inborn errors in primary bile acid synthesis responsive to treatment with cholic acid	Single	treatment of inborn errors of primary bile acid synthesis	20.9.2024	28.10.2009	EMA/OD/080/09	EU/3/09/683	Treatment of Inborn errors in primary bile acid synthesis responsive to treatment with cholic acid	
EMEA/H/C/000943	STANDARD	N	BioMarin International Limited	Kuvan	sapropterin	A16AX07	2.12.2008	2008	10.8.9999	VALID			GRANTED			Hyperphenylalaninemia	Single	treatment of hyperphenylalaninemia (HPA)	3.12.2020	8.6.2004	EMA/OD/077/03	EU/3/04/199	Treatment of Hyperphenylalaninemia	
EMEA/H/C/004090	STANDARD	N	Novartis Europharm Limited	Kymriah	tisagenlecleucel	L01	23.8.2018	2018	27.8.2023	VALID			GRANTED			B-lymphoblastic leukaemia/lymphoma	Gene therapy	treatment of B cell acute lymphoblastic leukaemia (ALL) and diffuse large B cell lymphoma (DLBCL)	26.8.2028	29.4.2014	EMA/OD/187/13	EU/3/14/1266	Treatment of B-lymphoblastic leukaemia/lymphoma	
EMEA/H/C/003790	STANDARD	Y	Amgen Europe B.V.	Kyprolis	carfilzomib	L01XX45	19.11.2015	2015	23.11.2020	VALID			GRANTED			Multiple myeloma	Peptide (chemically synthesised)	treatment of multiple myeloma	22.11.2025	3.6.2008	EMA/OD/120/07	EU/3/08/548	Treatment of Multiple myeloma	
EMEA/H/C/003922	EXCEPTIONAL	N	Chiesi Farmaceutici S.p.A.	Lamzede	velmanase alfa	A16AB15	23.3.2018	2018	27.3.2023	VALID			GRANTED			Alpha-mannosidosis	Recombinant proteins	indicated for long-term enzyme replacement therapy in patients with alpha-mannosidosis	26.3.2028	26.1.2005	EMA/OD/074/04	EU/3/04/260	Treatment of Alpha-mannosidosis	
EMEA/H/C/004216	CONDITIONAL	Y	Eli Lilly Nederland B.V.	Lartruvo	olaratumab	L01XC27	9.11.2016	2016	11.11.2019	REVOKED		23.7.2019	WITHDRAWN		Y	soft tissue sarcoma	Biologically derived proteins and peptides	treatment of soft tissue sarcoma		12.2.2015	EMA/OD/266/14	EU/3/15/1447	Treatment of soft tissue sarcoma	
EMEA/H/C/002826	STANDARD	N	Helsinn Birex Pharmaceuticals Limited	LEDAGA	chlormethine	L01AA05	3.3.2017	2017	7.3.2022	VALID			GRANTED			T-cell lymphoma	Chemicals	treatment of mycosis fungoides-type cutaneous T-cell lymphoma (MF-type CTCL)	6.3.2027	22.5.2012	EMA/OD/112/11	EU/3/12/963	Treatment of T-cell lymphoma	
EMEA/H/C/003727	STANDARD	Y	Eisai GmbH	Lenvima	lenvatinib	L01XE29	28.5.2015	2015	1.6.2020	VALID			EXPIRED			follicular thyroid cancer	Single	treatment of papillary, follicular thyroid cancer Treatment of follicular thyroid cancer	31.5.2025	26.4.2013	EMA/OD/019/13	EU/3/13/1119	Treatment of follicular thyroid cancer	
EMEA/H/C/000504	STANDARD	N	Lipomed GmbH	Litak	cladribine	L01BB04	14.4.2004	2004	10.8.9999	VALID			EXPIRED			Non-Hodgkin lymphoma	Single	treatment of hairy cell leukaemia	18.4.2014	18.9.2001	EMA/OD/006/01	EU/3/01/055	Treatment of Non-Hodgkin lymphoma	
EMEA/H/C/004123	STANDARD	N	Advanced Accelerator Applications	LUTATHERA	lutetium (177Lu) oxodotreotide	V10XX04	26.9.2017	2017	28.9.2022	VALID			GRANTED			gastro-entero-pancreatic neuroendocrine tumours	Radionuclides	treatment of gastro-entero-pancreatic neuroendocrine tumours	27.9.2027	31.1.2008	EMA/OD/093/07	EU/3/07/523	Treatment of gastro-entero-pancreatic neuroendocrine tumours	
EMEA/H/C/004451	STANDARD	N	Novartis Europharm Limited	Luxturna	voretigene neparvovec	S01XA27	22.11.2018	2018	5.12.2023	VALID			GRANTED			Leber's congenital amaurosis	Gene therapy	treatment of patients with vision loss due to Leber congenital amaurosis or retinitis pigmentosa inherited retinal dystrophy	4.12.2028	2.4.2012	EMA/OD/150/11	EU/3/12/981	Treatment of Leber's congenital amaurosis	
EMEA/H/C/003726	STANDARD	N	AstraZeneca AB	Lynparza	olaparib	L01XX46	16.12.2014	2014	10.8.9999	VALID			EXPIRED			ovarian cancer	Single	treatment of ovarian cancer and breast cancer	17.12.2024	6.12.2007	EMA/OD/063/07	EU/3/07/501	Treatment of ovarian cancer	
EMEA/H/C/000521	STANDARD	N	HRA Pharma Rare Diseases	Lysodren	mitotane	L01XX23	28.4.2004	2004	10.8.9999	VALID			EXPIRED			Adrenal cortical carcinoma	Single	treatment of adrenal cortical carcinoma	29.4.2014	12.6.2002	EMA/OD/003/02	EU/3/02/102	Treatment of Adrenal cortical carcinoma	
EMEA/H/C/000802	STANDARD	N	Takeda France SAS	Mepact	mifamurtide	L03AX15	6.3.2009	2009	10.8.9999	VALID		23.3.2019	EXPIRED		Y	Osteosarcoma	Peptide (chemically synthesised)	treatment of bone cancer		21.6.2004	EMA/OD/013/04	EU/3/04/206	Treatment of Osteosarcoma	
EMEA/H/C/004438	EXCEPTIONAL	N	Ultragenyx Germany GmbH	Mepsevii	vestronidase alfa	A16AB18	23.8.2018	2018	27.8.2023	VALID			GRANTED			mucopolysaccharidosis type VII (Sly syndrome)	Recombinant proteins	Mepsevii is indicated for the treatment of Mucopolysaccharidosis VII (MPS VII; Sly syndrome) for patients of all ages	26.8.2028	21.3.2012	EMA/OD/127/11	EU/3/12/973	Treatment of mucopolysaccharidosis type VII (Sly syndrome)	
EMEA/H/C/001030	STANDARD	N	Genzyme Europe BV	Mozobil	plerixafor	L03AX16	31.7.2009	2009	10.8.9999	VALID			GRANTED			Haematopoietic cell transplantation	Single	treatment of lymphoma and multiple myeloma	3.8.2019	20.10.2004	EMA/OD/045/04	EU/3/04/227	Treatment of Haematopoietic cell transplantation	
EMEA/H/C/004218	EXCEPTIONAL	N	Aegerion Pharmaceuticals B.V.	Myalepta	metreleptin	A16AA07	30.7.2018	2018	1.8.2023	VALID			GRANTED			familial partial lipodystrophy	Recombinant proteins	treatment of leptin deficiency (lipodystrophy)	31.7.2028	17.7.2012	EMA/OD/033/12	EU/3/12/1022	Treatment of familial partial lipodystrophy	
EMEA/H/C/004204	STANDARD	N	Pfizer Europe MA EEIG	Mylotarg	gemtuzumab ozogamicin	L01XC05	19.4.2018	2018	23.4.2023	VALID			GRANTED			acute myeloid leukaemia (AML).	Monoclonal antibodies	combination therapy with daunorubicin (DNR) and cytarabine (AraC) for the treatment of patients with previously untreated, de novo acute myeloid leukaemia (AML)	22.4.2028	18.10.2000	EMA/OD/022/00	EU/3/00/005	Treatment of acute myeloid leukaemia (AML).	
EMEA/H/C/000636	STANDARD	N	Genzyme Europe BV	Myozyme	alglucosidase alfa	A16AB07	29.3.2006	2006	10.8.9999	VALID		31.3.2016	EXPIRED			Glycogen storage disease type II (Pompe's disease)	Recombinant proteins	long-term enzyme replacement therapy (ERT) in patients with Pompe disease		14.2.2001		EU/3/01/018	Treatment of Glycogen storage disease type II (Pompe's disease)	
EMEA/H/C/000640	EXCEPTIONAL	N	BioMarin International Limited	Naglazyme	galsulfase	A16AB08	24.1.2006	2006	10.8.9999	VALID		26.1.2016	EXPIRED			Mucopolysaccharidosis, type VI (Maroteaux-Lamy Syndrome)	Recombinant proteins	enzyme replacement therapy of Mucopolysaccharidosis VI (MPS VI; N-acetylgalactosamine 4-sulfatase deficiency; Maroteaux-Lamy syndrome)		14.2.2001	EMA/OD/051/00	EU/3/01/025	Treatment of Mucopolysaccharidosis, type VI (Maroteaux-Lamy Syndrome)	
EMEA/H/C/004584	STANDARD	N	Lupin Europe GmbH	Namuscla	mexiletine	C01BB02	18.12.2018	2018	20.12.2023	VALID			GRANTED			acute myeloid leukaemia	Single	treatment of non-dystrophic myotonic disorders	19.12.2028	19.11.2014	EMA/OD/074/14	EU/3/14/1353	Treatment of acute myeloid leukaemia	
EMEA/H/C/003861	CONDITIONAL	N	Shire Pharmaceuticals Ireland Limited	Natpar	parathyroid hormone	H05AA03	24.4.2017	2017	26.4.2021	VALID			GRANTED			hypoparathyroidism	Recombinant proteins	treatment of hypoparathyroidism	25.4.2027	18.12.2013	EMA/OD/102/13	EU/3/13/1210	Treatment of hypoparathyroidism	
EMEA/H/C/000690	STANDARD	N	Bayer AG	Nexavar	sorafenib	L01XE05	19.7.2006	2006	10.8.9999	VALID		22.7.2016	EXPIRED			Renal cell carcinoma	Single	treatment of hepatocellular carcinoma, renal cell carcinoma and differentiated thyroid carcinoma		2.8.2004	EMA/OD/032/04	EU/3/04/207	Treatment of Renal cell carcinoma	
EMEA/H/C/002246	STANDARD	N	MediWound Germany GmbH	NexoBrid	concentrate of proteolytic enzymes enriched in bromelain	D03BA03	18.12.2012	2012	20.12.2022	VALID			GRANTED			Burns	Extracted proteins	removal of eschar	19.12.2022	30.7.2002	EMA/OD/012/02	EU/3/02/107	Treatment of Burns	
EMEA/H/C/003844	CONDITIONAL	N	Takeda Pharma A/S	NINLARO	ixazomib	L01XX50	21.11.2016	2016	23.11.2020	VALID			GRANTED			Multiple myeloma	Single	treatment of multiple myeloma	22.11.2026	27.9.2011	EMA/OD/048/11	EU/3/11/899	Treatment of Multiple myeloma	
EMEA/H/C/000942	STANDARD	N	Amgen Europe B.V.	Nplate	romiplostim	B02BX04	4.2.2009	2009	10.8.9999	VALID			EXPIRED			Thrombocytopenic purpura	Recombinant proteins	treatment of chronic immune (idiopathic) thrombocytopenic purpura (ITP)	5.2.2019	27.5.2005	EMA/OD/008/05	EU/3/05/283	Treatment of Thrombocytopenic purpura	
EMEA/H/C/004093	CONDITIONAL	N	Intercept Pharma International Limited	OCALIVA	obeticholic acid	A05AA04	12.12.2016	2016	15.12.2019	VALID			GRANTED			primary biliary cirrhosis	Single	treatment of primary biliary cirrhosis	14.12.2026	27.7.2010	EMA/OD/073/09	EU/3/10/753	Treatment of primary biliary cirrhosis	
EMEA/H/C/003821	STANDARD	Y	Boehringer Ingelheim International GmbH	OFEV	nintedanib	L01XE31	15.1.2015	2015	10.8.9999	VALID			GRANTED			idiopathic pulmonary fibrosis	Single	treatment of Idiopathic Pulmonary Fibrosis (IPF)	18.1.2025	26.4.2013	EMA/OD/186/12	EU/3/13/1123	Treatment of idiopathic pulmonary fibrosis	
EMEA/H/C/004125	STANDARD	N	Les Laboratoires Servier	Onivyde pegylated liposomal	irinotecan hydrochloride trihydrate	L01XX19	14.10.2016	2016	18.10.2021	VALID			GRANTED			pancreatic cancer	Single	treatment of pancreatic cancer	17.10.2026	9.12.2011	EMA/OD/051/11	EU/3/11/933	Treatment of pancreatic cancer	
EMEA/H/C/004699	STANDARD	Y	Alnylam Netherlands B.V.	Onpattro	patisiran	N07XX12	27.8.2018	2018	29.8.2023	VALID			GRANTED			familial amyloid polyneuropathy	RNA	treatment of hereditary transthyretin-mediated amyloidosis	28.8.2028	15.4.2011	EMA/OD/142/10	EU/3/11/857	Treatment of familial amyloid polyneuropathy	
EMEA/H/C/000466	EXCEPTIONAL	N	Pfizer Limited	Onsenal	celecoxib	L01XX33	17.10.2003	2003	16.10.2013	SURRENDERED			EXPIRED			Familial adenomatous polyposis (FAP)	Single	treatment of familial adenomatous polyposis	21.10.2013	20.11.2001	EMA/OD/011/01	EU/3/01/070	Treatment of Familial adenomatous polyposis (FAP)	
EMEA/H/C/002697	STANDARD	N	Janssen-Cilag International N.V.	Opsumit	macitentan	C02KX04	20.12.2013	2013	10.8.9999	VALID			GRANTED			pulmonary arterial hypertension	Single	treatment of pulmonary arterial hypertension (PAH)	26.12.2023	27.9.2011	EMA/OD/023/11	EU/3/11/909	Treatment of pulmonary arterial hypertension	
EMEA/H/C/000555	STANDARD	N	Swedish Orphan Biovitrum International AB	Orfadin	nitisinone	A16AX04	21.2.2005	2005	10.8.9999	VALID			EXPIRED			Tyrosinaemia type 2	Single	treatment of hereditary tyrosinemia type 1	23.2.2015	29.12.2000	EMA/OD/011/00	EU/3/00/012	Treatment of Tyrosinaemia type 2	
EMEA/H/C/001250	EXCEPTIONAL	N	Laboratoires CTRS	Orphacol	cholic acid	A05AA03	12.9.2013	2013	10.8.9999	VALID			GRANTED			Primary bile acid synthesis	Single	treatment of inborn errors in primary bile acid synthesis	15.9.2023	18.12.2002	EMA/OD/036/02	EU/3/02/127	Treatment of Primary bile acid synthesis	
EMEA/H/C/004209	STANDARD	Y	Dompe farmaceutici S.p.A.	OXERVATE	cenegermin	S01XA24	6.7.2017	2017	10.7.2022	VALID			GRANTED	4.20		neurotrophic keratitis	Recombinant proteins	treatment of neurotrophic keratitis	9.7.2027	14.12.2015	EMA/OD/143/15	EU/3/15/1586	Treatment of neurotrophic keratitis	
EMEA/H/C/004744	STANDARD	N	BioMarin International Limited	Palynziq	pegvaliase	A16AB19	3.5.2019	2019	8.5.2024	VALID			GRANTED			Hyperphenylalaninemia	Recombinant proteins	treatment of adults with phenylketonuria (PKU) who have inadequate blood phenylalanine control	7.5.2029	28.1.2010	EMA/OD/112/09	EU/3/09/708	Treatment of Hyperphenylalaninemia	
EMEA/H/C/000549	STANDARD	N	Recordati Rare Diseases	Pedea	ibuprofen	C01EB16	29.7.2004	2004	10.8.9999	VALID		2.8.2014	EXPIRED		Y	Patent ductus arteriosus	Single	treatment of patent ductus arteriosus		14.2.2001	EMA/OD/004/00	EU/3/01/020	Treatment of Patent ductus arteriosus	
EMEA/H/C/001014	STANDARD	N	Chiesi Farmaceutici S.p.A.	Peyona	caffeine citrate	N06BC01	2.7.2009	2009	10.8.9999	VALID		6.7.2019	EXPIRED		Y	primary apnoea of premature newborns	Single	treatment of primary apnoea		17.2.2003	EMA/OD/047/02	EU/3/03/132	Treatment of primary apnoea of premature newborns	
EMEA/H/C/000493	STANDARD	N	Pinnacle Biologics B.V.	PhotoBarr	porfimer sodium	L01XD01	25.3.2004	2004	24.3.2014	SURRENDERED			EXPIRED			Dysplasia in Barrett's oesophagus	Homopolymer	treatment of high-grade dysplasia (HGD) in Barrett's Esophagus (BE)	28.3.2014	6.3.2002	EMA/OD/065/01	EU/3/02/086	Treatment of Dysplasia in Barrett's oesophagus	
EMEA/H/C/002185	STANDARD	N	Shire Services BVBA	Plenadren	hydrocortisone	H02AB09	3.11.2011	2011	10.8.9999	VALID			GRANTED			Adrenal insufficiency	Single	treatment of adrenal insufficiency	13.11.2021	22.5.2006	EMA/OD/108/05	EU/3/06/372	Treatment of Adrenal insufficiency	
EMEA/H/C/004232	STANDARD	N	Kyowa Kirin Holdings B.V.	POTELIGEO	mogamulizumab	L01XC25	22.11.2018	2018	26.11.2023	VALID			GRANTED			cutaneous T-cell lymphoma	Monoclonal antibodies	treatment mycosis fungoides (MF) or Sézary syndrome (SS)	25.11.2028	14.10.2016	EMA/OD/091/16	EU/3/16/1756	Treatment of cutaneous T-cell lymphoma	
EMEA/H/C/004536	STANDARD	N	Merck Sharp & Dohme B.V.	PREVYMIS	letermovir	J05AX18	8.1.2018	2018	10.1.2023	VALID			GRANTED			Infection	Single	prophylaxis of cytomegalovirus (CMV) reactivation and disease	9.1.2028	15.4.2011	EMA/OD/090/10	EU/3/11/849	Prevention of Infection	
EMEA/H/C/000551	STANDARD	N	Riemser Pharma GmbH	Prialt	ziconotide	N02BG08	21.2.2005	2005	10.8.9999	VALID			EXPIRED			Pain	Peptide (chemically synthesised)	treatment of severe chronic pain	23.2.2015	9.7.2001	EMA/OD/062/00	EU/3/01/048	Treatment of Pain	
EMEA/H/C/002465	STANDARD	N	Chiesi Farmaceutici S.p.A.	Procysbi	mercaptamine	A16AA04	6.9.2013	2013	10.8.9999	VALID			GRANTED			cystinosis	Single	treatment of cystinosis	9.9.2023	20.9.2010	EMA/OD/034/10	EU/3/10/778	Treatment of cystinosis	
EMEA/H/C/003918	EXCEPTIONAL	N	EUSA Pharma (Netherlands) B.V.	Qarziba	dinutuximab beta	L01XC16	8.5.2017	2017	11.5.2022	VALID			GRANTED			neuroblastoma	Recombinant proteins	treatment of neuroblastoma	10.5.2027	8.11.2012	EMA/OD/112/12	EU/3/12/1062	Treatment of neuroblastoma	
EMEA/H/C/003822	STANDARD	N	Immedica Pharma AB	RAVICTI	glycerol phenylbutyrate	A16AX09	27.11.2015	2015	1.12.2020	VALID			GRANTED			Ornithine carbamoyltransferase deficiency	Single	treatment of patients with urea cycle disorders (UCD), ornithine transcarbamylase (OTC),argininosuccinate synthetase (ASS), arininosuccinate lyase (ASL), arginase I (ARG), CITRIN,ornithine translocase (HHH)	30.11.2025	10.6.2010	EMA/OD/002/10	EU/3/10/734	Treatment of Ornithine carbamoyltransferase deficiency	
EMEA/H/C/003834	EXCEPTIONAL	N	Santhera Pharmaceuticals (Deutschland) GmbH	Raxone	idebenone	N06BX13	8.9.2015	2015	10.9.2020	VALID			GRANTED			Neuropathy	Single	treatment of Leber's Hereditary Optic Neuropathy (LHON)	9.9.2025	15.2.2007	EMA/OD/076/06	EU/3/07/434	Treatment of Neuropathy	
EMEA/H/C/000369	STANDARD	N	Shire Human Genetic Therapies AB	Replagal	agalsidase alfa	A16AB03	3.8.2001	2001	10.8.9999	VALID			EXPIRED			Fabry disease	Recombinant proteins	treatment of Fabry disease	6.8.2011	8.8.2000	EMA/OD/007/00	EU/3/00/002	Treatment of Fabry disease	
EMEA/H/C/000638	STANDARD	N	Pfizer Europe MA EEIG	Revatio	sildenafil	G04BE03	28.10.2005	2005	10.8.9999	VALID			EXPIRED			Pulmonary arterial hypertension and chronic thromboembolic pulmonary hypertension	Single	treatment of pulmonary arterial hypertension	3.11.2015	12.12.2003	EMA/OD/056/03	EU/3/03/178	Treatment of Pulmonary arterial hypertension and chronic thromboembolic pulmonary hypertension	
EMEA/H/C/002345	STANDARD	N	Shire Pharmaceuticals Ireland Limited	Revestive	teduglutide	A16AX08	30.8.2012	2012	10.8.9999	VALID			GRANTED			Short Bowel Syndrome	Recombinant proteins	treatment of Short Bowel Syndrome	3.9.2022	11.12.2001	EMA/OD/045/01	EU/3/01/077	Treatment of Short Bowel Syndrome	
EMEA/H/C/000717	STANDARD	N	Celgene Europe BV	Revlimid	lenalidomide	L04AX04	14.6.2007	2007	14.6.2022	VALID			EXPIRED			multiple myeloma	Single	treatment of multiple myeloma, myelodysplastic syndromes and mantle cell lymphoma	18.6.2017	12.12.2003	EMA/OD/063/03	EU/3/03/177	Treatment of multiple myeloma	
EMEA/H/C/001110	STANDARD	N	Novartis Europharm Limited	Revolade	eltrombopag	B02BX05	11.3.2010	2010	10.8.9999	VALID			EXPIRED			Idiopathic thrombocytopenic purpura	Single	treatment of chronic idiopathic thrombocytopenic purpura (ITP)	14.3.2020	3.8.2007	EMA/OD/031/07	EU/3/07/467	Treatment of Idiopathic thrombocytopenic purpura	
EMEA/H/C/001047	EXCEPTIONAL	N	Regeneron UK Limited	Rilonacept Regeneron	rilonacept	L04AC04	23.10.2009	2009	22.10.2014	SURRENDERED			EXPIRED			Cryopirin-associated periodic syndromes	Recombinant proteins	treatment of Cryopyrin-Associated Periodic Syndromes (CAPS), including Familial Cold Autoinflammatory Syndrome (FCAS) and Muckle-Wells Syndrome (MWS)	26.10.2019	10.7.2007	EMA/OD/016/07	EU/3/07/456	Treatment of Cryopirin-associated periodic syndromes	
EMEA/H/C/004272	CONDITIONAL	N	Clovis Oncology Ireland Limited	Rubraca	rucaparib	L01XX55	24.5.2018	2018	29.5.2021	VALID		4.12.2018	EXPIRED			Ovarian cancer	Single	treatment of ovarian cancer		10.10.2012	EMA/OD/085/12	EU/3/12/1049	Treatment of Ovarian cancer	
EMEA/H/C/004095	STANDARD	N	Novartis Europharm Limited	Rydapt	midostaurin	L01XE39	18.9.2017	2017	20.9.2022	VALID			GRANTED			Acute myeloid leukaemia	Chemicals	treatment of mastocytosis and treatment of acute myeloid leukaemia	19.9.2027	29.7.2004	EMA/OD/028/04	EU/3/04/214	Treatment of Acute myeloid leukaemia	
EMEA/H/C/000682	STANDARD	N	Clinigen Healthcare B.V.	Savene	dexrazoxane	V03AF02	28.7.2006	2006	10.8.9999	VALID			EXPIRED			Anthracycline extravasations	Single	treatment of anthracycline extravasation	1.8.2016	19.9.2001	EMA/OD/021/01	EU/3/01/059	Treatment of Anthracycline extravasations	
EMEA/H/C/002548	EXCEPTIONAL	N	Clinuvel Europe Limited	SCENESSE	afamelanotide	D02BB02	22.12.2014	2014	10.8.9999	VALID			GRANTED			erythropoietic protoporphyria	Peptide (chemically synthesised)	treatment of phototoxicity	28.12.2024	8.5.2008	EMA/OD/108/07	EU/3/08/541	Treatment of erythropoietic protoporphyria	
EMEA/H/C/002052	STANDARD	N	Novartis Europharm Limited	Signifor	pasireotide	H01CB05	24.4.2012	2012	10.8.9999	VALID			GRANTED			acromegaly	Peptide (chemically synthesised)	treatment of Cushing's disease	20.11.2024	8.10.2009	EMA/OD/051/09	EU/3/09/670	Treatment of acromegaly	
EMEA/H/C/000689	STANDARD	N	Addmedica S.A.S.	Siklos	hydroxycarbamide	L01XX05	29.6.2007	2007	10.8.9999	VALID			EXPIRED			Sickle cell	Single	prevention of vaso-occlusive crises in patients with symptomatic Sickle Cell Syndrome	4.7.2017	9.7.2003	EMA/OD/013/03	EU/3/03/154	Treatment of Sickle cell	
EMEA/H/C/002614	CONDITIONAL	N	Janssen-Cilag International NV	SIRTURO	bedaquiline	J04AK05	5.3.2014	2014	7.3.2021	VALID			GRANTED			Tuberculosis	Single	treatement of pulmonary tuberculosis	6.3.2024	26.8.2005	EMA/OD/024/05	EU/3/05/314	Treatment of Tuberculosis	
EMEA/H/C/000791	STANDARD	Y	Alexion Europe SAS	Soliris	eculizumab	L04AA25	20.6.2007	2007	10.8.9999	VALID		22.6.2019	EXPIRED			paroxysmal nocturnal haemoglobinemia	Monoclonal antibodies	treatment of paroxysmal nocturnal haemoglobinuria / atypical haemolytic uremic syndrome / refractory generalized myasthenia gravis		17.10.2003	EMA/OD/042/03	EU/3/03/166	Treatment of paroxysmal nocturnal haemoglobinemia	
EMEA/H/C/004140	STANDARD	N	Advanced Accelerator Applications	SomaKit TOC	edotreotide	V09IX09	8.12.2016	2016	12.12.2021	VALID			GRANTED			gastro-entero-pancreatic neuroendocrine tumours	Peptide (chemically synthesised)	diagnosis of gastro-entero-pancreatic neuroendocrine tumours	11.12.2026	19.3.2015	EMA/OD/219/14	EU/3/15/1450	Diagnosis of gastro-entero-pancreatic neuroendocrine tumours	
EMEA/H/C/000409	STANDARD	N	Pfizer Europe MA EEIG	Somavert	pegvisomant	H01AX01	13.11.2002	2002	10.8.9999	VALID		15.11.2012	EXPIRED		Y	Acromegaly	Recombinant proteins	treatment of acromegaly		14.2.2001	EMA/OD/035/00	EU/3/01/023	Treatment of Acromegaly	
EMEA/H/C/004312	STANDARD	Y	Biogen Netherlands B.V.	Spinraza	nusinersen	M09AX07	30.5.2017	2017	1.6.2022	VALID			GRANTED			5q spinal muscular atrophy	RNA	treatment of Spinal Muscular Atrophy (SMA)	31.5.2029	2.4.2012	EMA/OD/141/11	EU/3/12/976	Treatment of 5q spinal muscular atrophy	
EMEA/H/C/000709	STANDARD	N	Bristol-Myers Squibb Pharma EEIG	Sprycel	dasatinib	L01XE06	20.11.2006	2006	10.8.9999	VALID			EXPIRED			Acute lymphoblastic leukaemia	Single	treatment of leukaemia	21.11.2016	23.12.2005	EMA/OD/074/05	EU/3/05/338	Treatment of Acute lymphoblastic leukaemia	

MAA Product Number	MA Authorisation Type (prod level)	Accelerated Review	MAA/MAH	Medicinal Product	INN	ATC Code	EU Birth date	EU Birth Year	Marketing Authorisation Valid Until Date	MAA Product Status	MA withdrawn date (other table)	Withdrawn /Expired Date	Orphan Status	Prevalence (per 10,000)	Significant Benefit	Broadest Disease Group	Type of Medicinal Product	Therapeutic Indication - Summary	Market Exclusivity Planned Expiry Date	Orphan Designation Date	Orphan Designation Procedure Number	Orphan Designation Number	Designated Orphan Indication	comment
EMEA/H/C/003794	EXCEPTIONAL	N	Alexion Europe SAS	Strensiq	asfotase alfa	A16AB13	28.8.2015	2015	1.9.2020	VALID			GRANTED			hypophosphatasia	Recombinant proteins	treatment of paediatric-onset hypophosphatasia	31.8.2025	3.12.2008	EMA/OD/071/08	EU/3/08/594	Treatment of hypophosphatasia	
EMEA/H/C/003854	STANDARD	N	Orchard Therapeutics (Netherlands) BV	Strimvelis	autologous CD34+ enriched cell fraction that contains CD34+ cells transduced with retroviral vector that encodes for the human ADA cDNA sequence	L03	26.5.2016	2016		VALID			GRANTED			Adenosine deaminase (ADA) deficiency	Gene therapy	severe combined immunodeficiency	29.5.2026	26.8.2005	EMA/OD/053/05	EU/3/05/313	Treatment of Adenosine deaminase (ADA) deficiency	
EMEA/H/C/003708	STANDARD	Y	EUSA Pharma (Netherlands) B.V.	SYLVANT	siltuximab	L04AC11	22.5.2014	2014	10.8.9999	VALID			GRANTED			Castleman's disease	Monoclonal antibodies	treatment of multicentric Castleman’s disease (MCD)	26.5.2024	30.11.2007	EMA/OD/078/07	EU/3/07/508	Treatment of Castleman's disease	
EMEA/H/C/004682	STANDARD	N	Vertex Pharmaceuticals (Ireland) Limited	Symkevi	tezacaftor / ivacaftor	R07AX31	31.10.2018	2018	6.11.2023	VALID			GRANTED			cystic fibrosis	Single	treatment of cystic fibrosis	5.11.2028	27.2.2017	EMA/OD/156/16	EU/3/17/1828	Treatment of cystic fibrosis	
EMEA/H/C/004806	STANDARD	Y	Shire Pharmaceuticals Ireland Limited	TAKHZYRO	lanadelumab	B06AC05	22.11.2018	2018	26.11.2023	VALID			GRANTED			hereditary angioedema	Monoclonal antibodies	prevention of angioedema attacks	25.11.2028	9.10.2015	EMA/OD/075/15	EU/3/15/1551	Treatment of hereditary angioedema	
EMEA/H/C/000798	STANDARD	N	Novartis Europharm Limited	Tasigna	nilotinib	L01XE08	19.11.2007	2007	10.8.9999	VALID		19.11.2019	EXPIRED		Y	Chronic myeloid leukaemia	Single	treatment of Philadelphia chromosome positive chronic myelogenous leukaemia (CML)		22.5.2006	EMA/OD/003/06	EU/3/06/375	Treatment of Chronic myeloid leukaemia	
EMEA/H/C/004782	STANDARD	Y	Akcea Therapeutics Ireland Limited	Tegsedi	inotersen	N07XX15	6.7.2018	2018	10.7.2023	VALID			GRANTED			Familial amyloid polyneuropathy	RNA	treatment of transthyretin amyloidosis (hATTR)	9.7.2028	26.3.2014	EMA/OD/098/13	EU/3/14/1250	Treatment of Familial amyloid polyneuropathy	
EMEA/H/C/001046	STANDARD	N	ADIENNE S.r.l.	TEPADINA	thiotepa	L01AC01	15.3.2010	2010	10.8.9999	VALID		17.3.2020	EXPIRED		Y	Conditioning treatment prior to haematopoietic progenitor cell transplantation	Single	conditioning treatment prior to conventional haematopoietic progenitor cell transplantation (HPCT)		29.1.2007	EMA/OD/060/06	EU/3/06/424	Conditioning treatment prior to haematopoietic progenitor cell transplantation	
EMEA/H/C/000823	STANDARD	N	Celgene Europe BV	Thalidomide Celgene	thalidomide	L04AX02	16.4.2008	2008	15.4.2023	VALID			EXPIRED			Multiple myeloma	Single	treatment of multiple myeloma	17.4.2018	20.11.2001	EMA/OD/040/01	EU/3/01/067	Treatment of Multiple myeloma	
EMEA/H/C/000679	STANDARD	N	Pfizer Limited	Thelin	sitaxentan sodium	C02KX03	10.8.2006	2006	10.8.2011	SURRENDERED			EXPIRED			Pulmonary arterial hypertension and chronic thromboembolic pulmonary hypertension	Single	treatment of idiopathic pulmonary arterial hypertension (IPAH) or pulmonary arterial hypertension	10.8.2016	21.10.2004	EMA/OD/031/04	EU/3/04/234	Treatment of Pulmonary arterial hypertension and chronic thromboembolic pulmonary hypertension	
EMEA/H/C/002155	STANDARD	N	Mylan IRE Healthcare Limited	TOBI Podhaler	tobramycin	J01GB01	20.7.2011	2011	10.8.9999	VALID			GRANTED			pulmonary infection due to Pseudomonas aeruginosa in cystic fibrosis	Single	long-term management of chronic pulmonary infection due to Pseudomonas aeruginosa	24.7.2023	17.3.2003	EMA/OD/072/02	EU/3/03/140	Treatment of pulmonary infection due to Pseudomonas aeruginosa in cystic fibrosis	
EMEA/H/C/000799	STANDARD	N	Pfizer Europe MA EEIG	Torisel	temsirolimus	L01XE09	19.11.2007	2007	10.8.9999	VALID			EXPIRED			Renal cell carcinoma	Single	treatment of renal cell carcinoma and mantle cell lymphoma	20.11.2017	6.4.2006	EMA/OD/103/05	EU/3/06/365	Treatment of Renal cell carcinoma	
EMEA/H/C/000401	STANDARD	N	Janssen-Cilag International NV	Tracleer	bosentan	C02KX01	15.5.2002	2002	10.8.9999	VALID		17.5.2012	EXPIRED		Y	Pulmonary arterial hypertension and chronic thromboembolic pulmonary hypertension	Single	treatment of pulmonary arterial hypertension and digital ulcers		14.2.2001	EMA/OD/036/00	EU/3/01/019	Treatment of Pulmonary arterial hypertension and chronic thromboembolic pulmonary hypertension	
EMEA/H/C/002720	CONDITIONAL	N	PTC Therapeutics International Limited	Translarna	ataluren	M09AX03	31.7.2014	2014	5.8.2020	VALID			GRANTED			Duchenne muscular dystrophy	Single	treatment of Duchenne muscular dystrophy.	4.8.2024	27.5.2005	EMA/OD/106/04	EU/3/05/278	Treatment of Duchenne muscular dystrophy	
EMEA/H/C/000388	STANDARD	N	Teva B.V.	TRISENOX	arsenic trioxide	L01XX27	5.3.2002	2002	10.8.9999	VALID		7.3.2012	EXPIRED		Y	Acute promyelocytic leukaemia	Single	treatment of relapsed acute promyelocytic leukaemia (APL)		18.10.2000	EMA/OD/008/00	EU/3/00/008	Treatment of Acute promyelocytic leukaemia	
EMEA/H/C/002800	STANDARD	N	United Therapeutics Europe Ltd	Unituxin	dinutuximab	L01XC16	14.8.2015	2015	18.8.2020	SURRENDERED			EXPIRED			Neuroblastoma	Monoclonal antibodies	treatment of neuroblastoma Treatment of high-risk neuroblastoma	17.8.2025	21.6.2011	EMA/OD/002/11	EU/3/11/879	Treatment of Neuroblastoma	
EMEA/H/C/004106	STANDARD	N	AbbVie Deutschland GmbH & Co. KG	Venclyxto	venetoclax	L01XX52	5.12.2016	2016	7.12.2024	VALID			EXPIRED			chronic lymphocytic leukaemia	Single	treatment of adult patients with chronic lymphocytic leukaemia (CLL)	6.12.2026	6.12.2012	EMA/OD/124/12	EU/3/12/1080	Treatment of chronic lymphocytic leukaemia	
EMEA/H/C/000474	STANDARD	N	Bayer AG	Ventavis	iloprost	B01AC11	16.9.2003	2003	10.8.9999	VALID		18.9.2013	EXPIRED		Y	Pulmonary arterial hypertension and chronic thromboembolic pulmonary hypertension	Single	treatment of primary pulmonary hypertension		29.12.2000	EMA/OD/009/00	EU/3/00/014	Treatment of Pulmonary arterial hypertension and chronic thromboembolic pulmonary hypertension	
EMEA/H/C/004411	STANDARD	Y	Santen Oy	Verkazia	ciclosporin	S01XA18	6.7.2018	2018	10.7.2023	VALID			GRANTED			vernal keratoconjunctivitis	Single	treatment of severe vernal keratoconjunctivitis (VKC)	9.7.2028	6.4.2006	EMA/OD/106/05	EU/3/06/360	Treatment of vernal keratoconjunctivitis	
EMEA/H/C/000978	STANDARD	N	Celgene Europe BV	Vidaza	azacitidine	L01BC07	17.12.2008	2008	10.8.9999	VALID			EXPIRED			Myelodysplastic syndromes	Single	Treatment of myelodysplastic syndromes (MDS), chronic myelomonocytic leukemia (CMML) and acute myeloid leukemia (AML)	21.12.2018	6.2.2002	EMA/OD/059/01	EU/3/01/084	Treatment of Myelodysplastic syndromes	
EMEA/H/C/002779	STANDARD	N	BioMarin International Limited	Vimizim	elosulfase alfa	A16AB12	28.4.2014	2014	10.8.9999	VALID			GRANTED			Morquio A syndrome	Recombinant proteins	treatment of mucopolysaccharidosis	29.4.2024	24.7.2009	EMA/OD/017/09	EU/3/09/657	Treatment of Morquio A syndrome	
EMEA/H/C/000839	STANDARD	N	GlaxoSmithKline (Ireland) Limited	Volibris	ambrisentan	C02KX02	21.4.2008	2008	10.8.9999	VALID			EXPIRED			pulmonary arterial hypertension and chronic thromboembolic pulmonary hypertension	Single	treatment of pulmonary arterial hypertension (PAH)	23.4.2018	11.4.2005	EMA/OD/103/04	EU/3/05/273	Treatment of pulmonary arterial hypertension and chronic thromboembolic pulmonary hypertension	
EMEA/H/C/002311	STANDARD	N	Novartis Europharm Limited	Votubia	everolimus	L01XE10	2.9.2011	2011	23.11.2020	VALID			GRANTED			Tuberous sclerosis complex	Single	treatment of subependymal giant cell astrocytoma (SEGA) associated with tuberous sclerosis (TS)	5.9.2021	4.8.2010	EMA/OD/010/10	EU/3/10/764	Treatment of Tuberous sclerosis complex	
EMEA/H/C/001249	STANDARD	Y	Shire Pharmaceuticals Ireland Limited	VPRIV	velaglucerase alfa	A16AB10	26.8.2010	2010	25.8.2020	VALID			GRANTED			Gaucher disease	Recombinant proteins	treatment of type 1 Gaucher disease	29.8.2022	9.6.2010	EMA/OD/140/09	EU/3/10/752	Treatment of Gaucher disease	
EMEA/H/C/002294	EXCEPTIONAL	N	Pfizer Europe MA EEIG	Vyndaqel	tafamidis	N07XX08	16.11.2011	2011	10.8.9999	VALID			GRANTED			familial amyloid polyneuropathy	Single	treatment of transthyretin amyloidosis in adult patients with cardiomyopathy (ATTR-CM)	17.11.2021	28.8.2006	EMA/OD/032/06	EU/3/06/401	Treatment of familial amyloid polyneuropathy	
EMEA/H/C/004282	STANDARD	N	Jazz Pharmaceuticals Ireland Limited	Vyxeos liposomal	daunorubicin / cytarabine	L01XY01	23.8.2018	2018	23.8.2023	VALID			GRANTED			acute myeloid leukaemia	Single	treatment of adults with high-risk acute myeloid leukaemia (AML)	26.8.2028	11.1.2012	EMA/OD/070/11	EU/3/11/942	Treatment of acute myeloid leukaemia	
EMEA/H/C/002616	STANDARD	N	BIOPROJET PHARMA	Wakix	pitolisant	N07XX11	31.3.2016	2016	4.4.2021	VALID			GRANTED			Narcolepsy	Chemicals	treatment of narcolepsy	3.4.2026	10.7.2007	EMA/OD/087/06	EU/3/07/459	Treatment of Narcolepsy	
EMEA/H/C/004538	CONDITIONAL	N	Akcea Therapeutics Ireland Limited	WAYLIVRA	volanesorsen	C10AX	3.5.2019	2019	8.5.2020	VALID			GRANTED	0.10		familial chylomicronemia syndrome	Peptide (chemically synthesised)	treatment of patients with familial chylomicronemia syndrome (FCS)	7.5.2029	19.2.2014	EMA/OD/180/13	EU/3/14/1249	Treatment of familial chylomicronemia syndrome	
EMEA/H/C/000535	STANDARD	N	Recordati Rare Diseases	Wilzin	zinc	A16AX05	13.10.2004	2004	10.8.9999	VALID			EXPIRED			Wilson’s disease	Single	treatment of Wilson's disease	17.10.2014	31.7.2001	EMA/OD/006/00	EU/3/01/050	Treatment of Wilson’s disease	
EMEA/H/C/000480	STANDARD	N	Shire Pharmaceuticals Ireland Limited	Xagrid	anagrelide	L01XX35	16.11.2004	2004	10.8.9999	VALID		18.11.2016	EXPIRED		Y	Essential thrombocythaemia	Single	reduction of elevated platelet counts in at risk essential thrombocythaemia patients		29.12.2000	EMA/OD/013/00	EU/3/00/010	Treatment of Essential thrombocythaemia	
EMEA/H/C/002022	STANDARD	N	Nova Laboratories Ireland Limited	Xaluprine	mercaptopurine	L01BB02	9.3.2012	2012	13.3.2022	VALID			GRANTED			acute lymphoblastic leukaemia	Single	treatment of acute lymphoblastic leukaemia (ALL)	12.3.2022	30.4.2009	EMA/OD/114/08	EU/3/09/628	Treatment of acute lymphoblastic leukaemia	
EMEA/H/C/003937	STANDARD	N	Ipsen Pharma	Xermelo	telotristat ethyl	A16AX15	18.9.2017	2017	20.9.2022	VALID			GRANTED			carcinoid tumours	Single	treatment of carcinoid syndrome	19.9.2027	8.10.2009	EMA/OD/047/09	EU/3/09/661	Treatment of carcinoid tumours	
EMEA/H/C/004752	STANDARD	Y	Astellas Pharma Europe B.V.	XOSPATA	gilteritinib	L01XE54	24.10.2019	2019		VALID			GRANTED			acute myeloid leukaemia	Chemicals	treatment of patients who have relapsed or refractory acute myeloid leukemia (AML) with a FLT3 mutation		17.1.2018	EMA/OD/175/17	EU/3/17/1961	Treatment of acute myeloid leukaemia	
EMEA/H/C/004480	STANDARD	N	Kite Pharma EU B.V.	Yescarta	axicabtagene ciloleucel	L01X	23.8.2018	2018	27.8.2023	VALID			GRANTED			diffuse large B cell lymphoma	Autologous (Genetically modified cell)	treatment of diffuse large B-cell lymphoma (DLBCL), primary mediastinal B-cell lymphoma (PMBCL) and transformed follicular lymphoma (TFL)	26.8.2028	16.12.2014	EMA/OD/171/14	EU/3/14/1393	Treatment of diffuse large B cell lymphoma	
EMEA/H/C/000773	STANDARD	N	Pharma Mar, S.A.	Yondelis	trabectedin	L01CX01	17.9.2007	2007	10.8.9999	VALID			EXPIRED			Soft tissue sarcoma	Single	treatment of soft tissue sarcoma and ovarian cancer	19.9.2017	30.5.2001	EMA/OD/001/01	EU/3/01/039	Treatment of Soft tissue sarcoma	
EMEA/H/C/002801	CONDITIONAL	N	MolMed S.p.A	Zalmoxis	nalotimagene carmaleucel	L01	18.8.2016	2016	23.8.2019	SURRENDERED		11.10.2019	WITHDRAWN		Y	Adjunctive treatment in haematopoietic cell transplantation	Allogeneic (Cell therapy)	treatment in haploidentical haematopoietic stem cell transplantation		20.10.2003	EMA/OD/041/03	EU/3/03/168	Adjunctive treatment in haematopoietic cell transplantation	
EMEA/H/C/000435	STANDARD	N	Janssen-Cilag International N.V.	Zavesca	miglustat	A16AX06	20.11.2002	2002	10.8.9999	VALID		21.11.2012	EXPIRED		Y	Gaucher disease	Single	treatment of Gaucher disease and Niemann-Pick type C disease		18.10.2000	EMA/OD/023/00	EU/3/00/006	Treatment of Gaucher disease	
EMEA/H/C/004249	STANDARD	N	GlaxoSmithKline (Ireland) Limited	Zejula	niraparib	L01XX54	16.11.2017	2017	20.11.2022	VALID			GRANTED			ovarian cancer	Single	treatment of epithelial ovarian, fallopian tube, or primary peritoneal cancer	19.11.2027	4.8.2010	EMA/OD/015/10	EU/3/10/760	Treatment of ovarian cancer	
EMEA/H/C/003691	CONDITIONAL	Y	bluebird bio (Netherlands) B.V	Zynteglo	betibeglogene autotemcel	B06	29.5.2019	2019	4.6.2020	VALID			GRANTED			beta-thalassemia intermedia and major	Autologous (Genetically modified cell - Gene therapy)	treatment of transfusion-dependent β-thalassaemia (TDT)	3.6.2029	24.1.2013	EMA/OD/146/12	EU/3/12/1091	Treatment of beta-thalassemia intermedia and major	
